# The Potential for Physiological Performance Curves to Shape Environmental Effects on Social Behavior

**DOI:** 10.3389/fphys.2021.754719

**Published:** 2021-11-11

**Authors:** Shaun S. Killen, Daphne Cortese, Lucy Cotgrove, Jolle W. Jolles, Amelia Munson, Christos C. Ioannou

**Affiliations:** ^1^Institute of Biodiversity, Animal Health and Comparative Medicine, University of Glasgow, Glasgow, United Kingdom; ^2^Center for Ecological Research and Forestry Applications (CREAF), Campus de Bellaterra (UAB), Barcelona, Spain; ^3^School of Biological Sciences, University of Bristol, Bristol, United Kingdom

**Keywords:** physiology, environmental change, individual heterogeniety, individual differences, phenotypic plasticity, social grouping, individual variation

## Abstract

As individual animals are exposed to varying environmental conditions, phenotypic plasticity will occur in a vast array of physiological traits. For example, shifts in factors such as temperature and oxygen availability can affect the energy demand, cardiovascular system, and neuromuscular function of animals that in turn impact individual behavior. Here, we argue that nonlinear changes in the physiological traits and performance of animals across environmental gradients—known as physiological performance curves—may have wide-ranging effects on the behavior of individual social group members and the functioning of animal social groups as a whole. Previous work has demonstrated how variation between individuals can have profound implications for socially living animals, as well as how environmental conditions affect social behavior. However, the importance of variation between individuals in how they respond to changing environmental conditions has so far been largely overlooked in the context of animal social behavior. First, we consider the broad effects that individual variation in performance curves may have on the behavior of socially living animals, including: (1) changes in the rank order of performance capacity among group mates across environments; (2) environment-dependent changes in the amount of among- and within-individual variation, and (3) differences among group members in terms of the environmental optima, the critical environmental limits, and the peak capacity and breadth of performance. We then consider the ecological implications of these effects for a range of socially mediated phenomena, including within-group conflict, within- and among group assortment, collective movement, social foraging, predator-prey interactions and disease and parasite transfer. We end by outlining the type of empirical work required to test the implications for physiological performance curves in social behavior.

## Introduction

Within species there exists considerable among-individual variation in numerous physiological traits associated with energy demand (Burton et al., 2011; Metcalfe et al., 2016), cardiorespiratory systems (Walsberg et al., 1986; Brijs et al., 2019), and neuromuscular function and movement (Wilson et al., 2004; Marras et al., 2010). A major aim in the field of ecophysiology is to understand how these traits are linked with organismal performance and behavior in an ecological context, including the ability to escape predators and obtain resources (Jablonszky et al., 2017; Mathot et al., 2017; Killen et al., 2017a). More recently, there has been growing interest in how among-individual heterogeneity in physiological traits can modulate animal social behavior, including social hierarchies (Kochhann, 2017), social networks (Moyers et al., 2018), and emergent collective behavior and group functioning (Jolles et al., 2017, 2020a). While there is a growing appreciation for the physiological underpinnings of social behavior (Seebacher and Krause, 2017), a more nuanced understanding of how environmental factors influence differences between individuals across a gradient is needed to accurately predict how social groups will respond to changing environments.

Social grouping ranges from pairs of animals to large scale communities and enormous aggregations consisting of millions of individuals. Variation in this tendency to group, both at the individual and species level, can be explained by the balance between the benefits of reducing predation risk, improving foraging and saving energy during locomotion, vs. the costs of competition within groups over food and the opportunity to breed, and a greater exposure to socially-transmitted diseases. These benefits and costs can be shifted, however, by individuals’ behavior within groups, with effects on social interactions and group functioning (Jolles et al., 2017; del Mar Delgado et al., 2018). However, increasing evidence suggests that social behavior is also related to physiological traits associated with individual’s metabolic phenotype (Killen et al., 2017b; Cooper et al., 2018), stress responsiveness (Spencer, 2017), cognition (Wascher et al., 2018), locomotor performance and movement speed (Jolles et al., 2017; Hansen et al., 2020), and immune function (Raulo et al., 2018). Physiological traits associated with bioenergetics and locomotion may be especially important in this regard because they are sensitive to environmental factors and can also influence performance in a social context, affecting both the capacity and motivation to express various behaviors. Metabolic rate, for example, has been linked with dominance and risk-prone behaviors (Mathot et al., 2019), which in turn have links with individual sociability (Jolles et al., 2017). There is also evidence of direct links between metabolic demand and sociability, with individuals with a higher metabolic rate being perhaps less social and therefore less likely to associate with conspecifics (Killen et al., 2016b; Cooper et al., 2018; but see Killen et al., 2021).

Social interactions can be influenced by environmental factors such as food abundance and potential predation risk (Beauchamp, 2004; Schaerf et al., 2017), but also by many aspects of the abiotic environment, including light levels (Ginnaw et al., 2020), temperature (Bartolini et al., 2015), hypoxia (Domenici et al., 2017), turbidity (Chamberlain and Ioannou, 2019), and habitat structure (Takada and Minami, 2021), as well as by anthropogenic changes such as acoustic noise (Currie et al., 2020), and pollutants (Armstrong et al., 2011). While environmental factors can impact behavior through the masking of cues and signals (McNett et al., 2010) and shifting attention to other tasks (Chan et al., 2010), environmental conditions can also affect behavior *via* physiological changes. The effects of environmental variables on social behavior *via* physiological changes can be indirect by inducing stress *via* stress hormones, or can directly affect the physiological traits associated with locomotor performance and movement speed, such as muscular function and aerobic and anaerobic capacity (Ord and Stamps, 2017). Because movement speed plays a fundamental role in leadership, cohesion, and alignment (Pettit et al., 2015; Jolles et al., 2020b), these aspects of social behavior may be sensitive to environmental perturbations. Over various timescales, changing environmental conditions will influence physiological trait expression, which will in turn affect social behavior and the degree of among-individual trait variation and trait repeatability (Killen et al., 2016a; Huang et al., 2020). These effects of environmental conditions on social behavior are becoming increasingly important to understand due to human-induced rapid environmental change (Sih, 2013; Barrett et al., 2019; Fisher et al., 2021).

**Figure 1:**
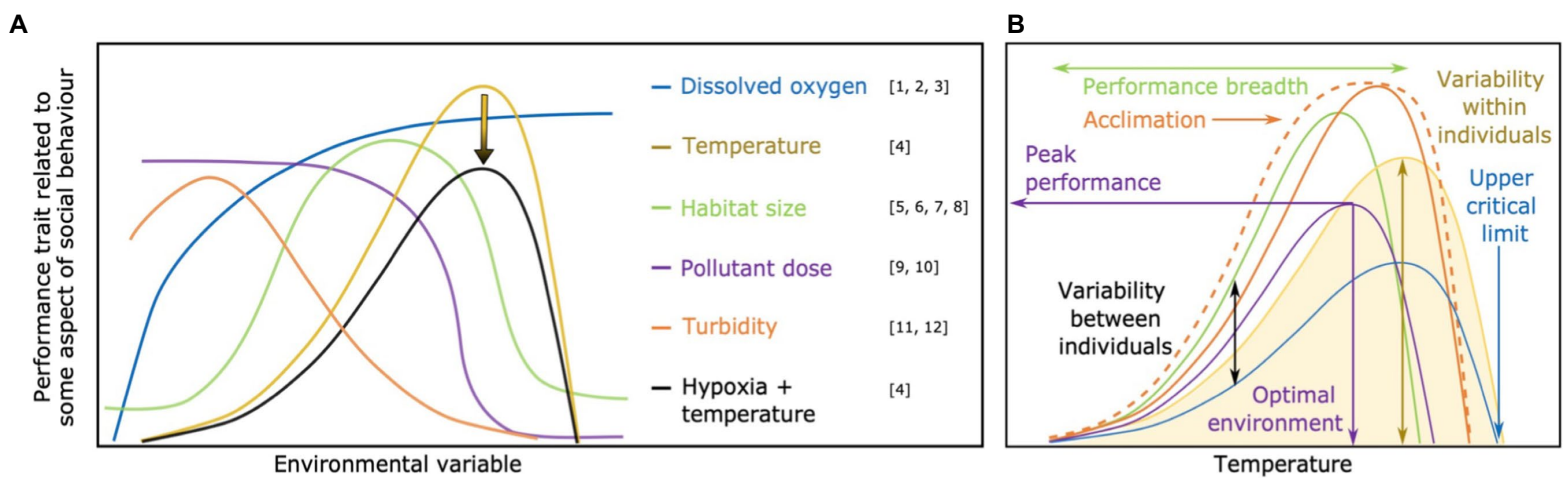
**(A)** Performance curve shape is heavily dependent on the environmental factor being examined. In this panel, different types of environmental factors are represented by different colors. The arrow represents an overall depression of trait expression when potential effects of hypoxia are combined with the effects of temperature. Note, when habitat size increases, greater protection/space to hide from predators and/or increase food availability may enhance performance, thus reducing endocrine stress level (Breves and Specker, 2005; Bauer et al., 2013). However, when territory is very large the performance traits may be reduced again in territorial animals (e.g., anemonefish; Ross, 1978) due to increased stress and/or energy investment to protect a larger area from competitors or predators. **(B)** Potential effects of among-individual variation in performance curves for a trait related to the expression of social behavior (e.g., aerobic capacity, cognitive ability, locomotor capacity, muscular function) in response to temperature (environmental variable). In this panel, the performance curve of different individuals within a social group are represented in different colors. The dashed orange line shows variation in the performance curve (solid orange line) caused by acclimation to the environmental variable (temperature in this example). Acclimation generally results in an overall “flattening” of the performance curve, but may also cause an increase in the peak performance. Arrows illustrate the different points of individual variation in performance curve that have implications for animal social behavior, especially in ectotherms. Each point and its consequence on social behavior is highlighted in Figures 2–7. References: [1] Barrionuevo and Burggren (1999); [2] Fry (1971); [3] Pörtner (2010); [4] Pörtner and Farrell (2008); [5] Maierdiyali et al. (2020); [6] Bauer et al. (2013); [7] Breves and Specker (2005); [8] Ross (1978); [9] Gomez Isaza et al. (2020); [10] McKenzie et al. (2007); [11] Meager et al. (2006); [12] Chamberlain and Ioannou (2019).

Breakthroughs in our understanding of the mechanistic underpinnings of sociality could be facilitated by studying the effects of individual performance curves on social dynamics. Functionally, performance curves are a type of reaction norm, but specifically depict shifts in fitness or a putative fitness proxy—often a physiological variable—across a continuous gradient of an environmental variable (as opposed to discrete measurements at specific environmental conditions), and generally exclude developmental effects on phenotypes to instead focus on relatively recent changes in environmental conditions (Kingsolver et al., 2014). Performance curves are therefore usually nonlinear—though they may appear linear within narrow environmental ranges—with their exact shape depending on the trait and environmental variable being considered (Kingsolver et al., 2014; Figure 1A). Such curves are generally determined for specific physiological traits or performance indices, expressed as biological rates, such as maximum locomotor speed or aerobic capacity, with performance defined as the capacity to express a given trait across a range of environmental conditions. As an example, in ectotherms a typical performance curve for maximum locomotor speed would be a gradual increase with temperature, a peak level of performance at an optimal temperature, followed by a decline in performance capacity with further warming (yellow line in Figure 1A). Performance curves often depict the change of a physiological trait in response to the environment and can therefore reflect environmental sensitivity (Kingsolver and Gomulkiewicz, 2003; Lefevre, 2016; Jutfelt et al., 2018). This sensitivity may, in turn, affect the capacity or motivation to perform specific behaviors, but these links are often uncertain and the focus of study to provide insight into intra- and intergenerational responses to environmental stressors (Metcalfe et al., 2016; Norin and Metcalfe, 2019).

Here, we argue that performance curves, and especially individual variation in performance curves within groups (Figure 1B), may be key in understanding how social behaviors are affected by shifting environmental conditions. In their natural environment, socially grouping animals can experience environmental changes at a scale of minutes, days, or months, but will also experience environmental changes over more protracted timeframes in response to broadscale phenomena such as climate change. For example, many animal species accommodate seasonal changes in temperature that are consistent across years, but due to human-induced climate change, such changes are becoming more extreme (IPCC, 2019). A more mechanistic, physiologically-based approach to the study of social behavior will be key for understanding how routine environmental shifts affect social behaviors as well as predict how social behavior may change or evolve in response to anthropogenic disturbances.

The study of animal social systems and particularly the study of collective behavior has transitioned from a focus on uncovering universal mechanisms underpinning emergent behavior and self-organization (Couzin et al., 2002), to an increasing recognition that among-individual heterogeneity plays a critical role in these processes (del Mar Delgado et al., 2018; Jolles et al., 2020a). We suggest that a promising next step in this line of research will be to examine how the degree of heterogeneity *itself* can change depending on the environment—as is dictated by individual performance curves—and how this will influence various dimensions of animal social behavior. We first discuss the broad effects that individual variation in performance curves within social groups may have on the relative physiological capacity and behavioral motivation of individuals within social groups. Next, we discuss the specific consequences of these effects for an array of ecological phenomena related to social behavior including within-group conflict, leader-follower dynamics, predator avoidance, and social foraging. Our aim is to highlight the enormous potential for performance curves to alter social behavior at the individual, group, and community level and outline priority areas for future research.

## Individual Variation in Performance Curves

A key factor to consider when assessing the impact of performance curves on social behavior is among-individual variation in how animals physiologically respond to changes in their environment (Bulté and Blouin-Demers, 2006). For example, different individuals can show different physiological sensitivities to factors such as temperature (Navas et al., 1999), or requirements in terms of oxygen (Killen et al., 2012b; Pang et al., 2015) or nutrition (Killen et al., 2011), with direct effects on among-individual variation in bioenergetics and capacity for locomotor performance. Such variation has traditionally been examined in the context of reaction norms whereby individuals are repeatedly measured for traits at around 2 or 3 environmental levels and modelled using mixed-models with random slopes (Dingemanse et al., 2010). However, assumptions of linearity may not be appropriate for all traits and particularly over broader environmental ranges. Therefore, to properly assess intra-individual variation in environmental sensitivity, the assessment of individual performance curves may be required (Gilbert and Miles, 2017). This work is still in its infancy, but investigations to date indicate that, similar to the case with linear reaction norms (van de Pol, 2012; Roche et al., 2016), individuals within species show variation in performance curves (Careau et al., 2014; Bartheld et al., 2017; Childress and Letcher, 2017; Nowakowski et al., 2020). There is also evidence that there may be within-individual variation in performance curves, in response to factors such as recent feeding history (Gilbert and Miles, 2016), which adds an extra layer of complexity.

If individual animals show variable degrees of behavioral and physiological plasticity in response to environmental variables, this has a wide range of potential consequences for social behavior. To illustrate this, consider among-individual variability in performance curves for a physiological trait (e.g., aerobic capacity or optimum movement speed) relevant to social behavior, in relation to some environmental variable (e.g., temperature; Figure 1B; van Berkum, 1988). There are numerous effects that emerge from individual variation in environmental sensitivity that could have important consequences for how individuals interact with each other within social groups, which we discuss in detail below. Important to consider for any of these effects is the influence of acclimation to environmental conditions. During acute environmental changes, such as in temperature or oxygenation, individual animals tend to show much stronger changes in the expression of their physiology or behavior (Guderley, 1990). These responses generally dampen with physiological acclimation to the new conditions (i.e., specific points along a performance curve), resulting in an overall “flattening” of the performance curve. Depending on the acclimation response of each individual groupmate and on the timescale of exposure to a given environment, the relative importance of each of the following considerations may change in prominence.

### Changes in the Rank Order of Performance Capacity

**Figure 2:**
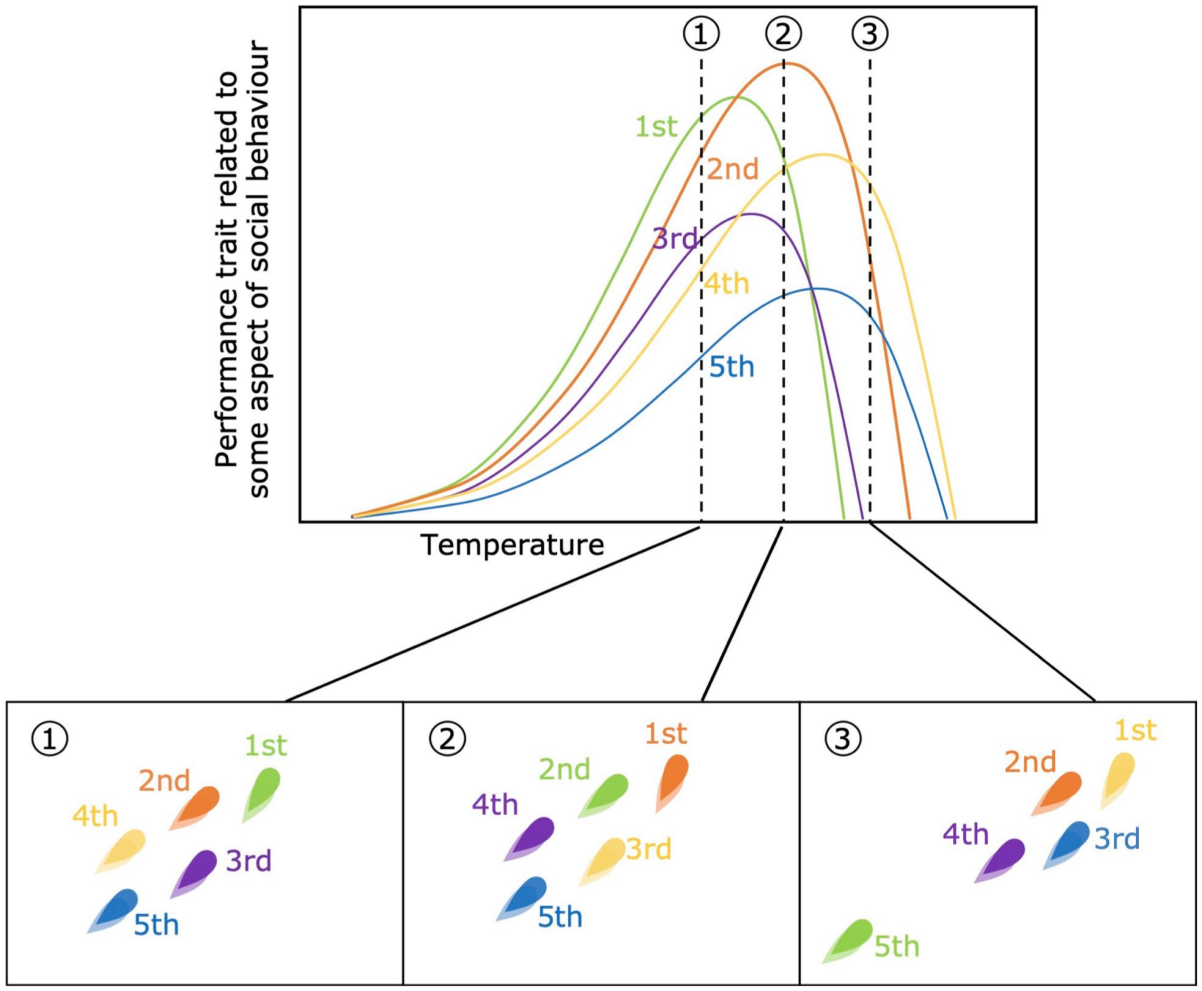
Changes in the rank order of performance capacity across three different temperatures (top panel). Each color refers to an individual within the same social group. In the bottom panels the rank-assortment within the group is shown for each temperature (1, 2 and 3), assuming that higher-ranked individuals are positioned on the front of the group. For example, the green individual is the highest rank-individual (leader) at temperature 1, but a follower with 2nd rank position at temperature 2, and is no longer part of the group at temperature 3, given that the individual’s performance capacity decreases to 0 before temperature 3, while the rest of the groups has not.

Differences in sensitivity to the environmental variable in question may generate differences in the rank order of performance capacity among individuals within a social group that directly depends on where along the environmental gradient performance is being measured (Figure 2). All else being equal, differences in this rank order could mean that, for example, the individual most likely to be dominant or a leader at one temperature may be subordinate or a follower at another temperature. Aside from having a direct effect on the social behaviors displayed by individuals, changes in the rank order of traits will also decrease their repeatability and, potentially, the ability of that trait to be a target for selection in a social context. Another key consideration is that, if relative differences in energy demand (related to food-acquisition) or locomotor ability (related to predator avoidance) change among individuals, then the fundamental costs and benefits of sociality and group membership could change differently for individual group members depending on the current environmental conditions (Cooper et al., 2018). As an example, at higher temperatures all individuals may be expected to become less social due to an increased escape ability, *via* increased muscle contractile ability and nervous stimulation (Johnson and Bennett, 1995), and decreased motivation to share or compete for discovered resources. Thereby differences in the steepness of individual performance curves could influence when specific individuals no longer benefit from staying in a group.

### Change in Among-Individual Variation

**Figure 3:**
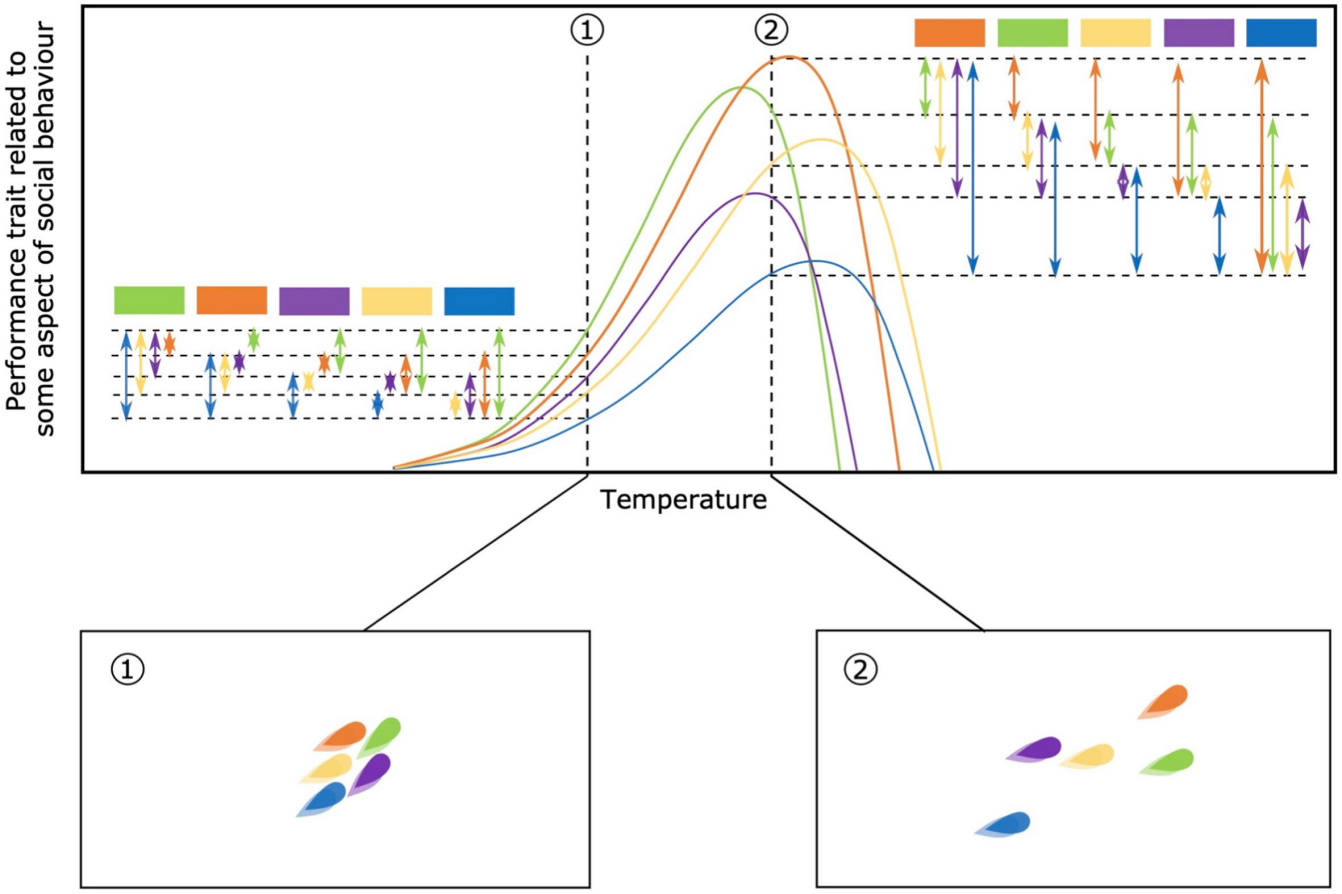
Change in among-individual variation along the environmental gradient. On the top panel the among-individual variation is highlighted at 2 different temperatures, when individual performance curves converge (temperature 1) or diverge (temperature 2). Each color refers to an individual within the same social group. Square boxes represent individuals used as a reference to show the amount of variation. Arrows show the amount of variation between individuals. In the bottom panels is shown an example of the consequences of among-individual variation in performance curves on social groups. Wider variation could lead to less cohesion, i.e., higher distances among individuals within the same group, here shown at temperature 2 compared to temperature 1.

As individual performance curves diverge or converge along the environmental gradient, the amount of phenotypic variation among individuals will correspondingly change. At a low temperature, for example, there may be a modest degree of among-individual variation in movement speed while at a higher temperature there may be wider variation (Killen et al., 2013; Figure 3). This change in the degree of variation among-individuals within a social group could have consequences for group coordination, cohesion, or intra-group conflict (Jolles et al., 2020a). Changing environmental conditions and among-individual variation may therefore cause groups to split or merge, which in turn may impact the degree of phenotypic differences among groups. In particular, at environmental extremes, suitable habitats may become scarce, causing groups to merge and potentially homogenize. Importantly, changes in the amount of among-individual variation are fundamental in exposing traits to selective pressures in the social context (Farine et al., 2015).

### Change in Within-Individual Variation

**Figure 4:**
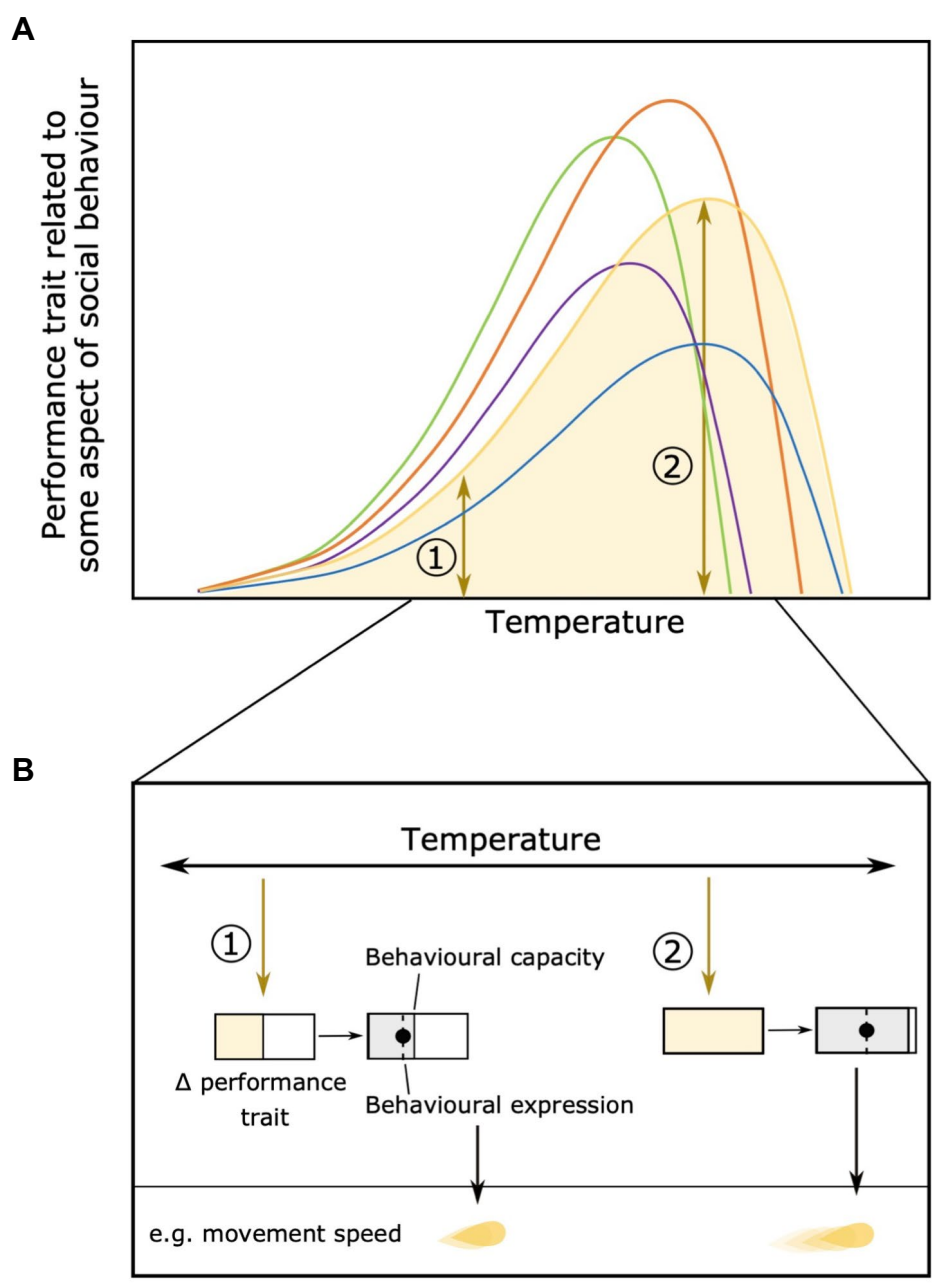
Change in within-individual variation across an environmental gradient (e.g., temperature). The area below the performance curve indicates the variation in individual performance [**(A)**, individual in yellow used as an example]. Differences in individual variation in performance trait at two different temperatures [1 and 2 **(B)**] can result in different behavioral capacity and expression. For example, at temperature 1 the yellow individual has only little variation in performance and its behavior is only expressed as low movement speed, while at temperature 2 (close to its optimum) the same individual has a higher variation of movement and can move up to very high speeds. Panel **(B)** reproduced from Jolles et al. (2020a).

The effects of environmental conditions on variation among individuals may extend to physiological and behavioral flexibility within individuals. Depending on the environment, individuals may become more or less flexible in their behavioral expression. Physiological constraints at very low or high temperatures, for example, may limit the behavioral options available to individuals. At temperatures around their individual optimum, however, individuals should be less constrained and more able to express behavior based on moment-to-moment changes in their motivation (Jolles et al., 2020a; Figure 4). Changes in within-individual variation along performance curves could also have consequences for the ability of natural selection to act on that trait if there are changes in across- or within-context trait repeatability (Killen et al., 2016a).

### Among-Individual Differences in Optimal Environments

**Figure 5:**
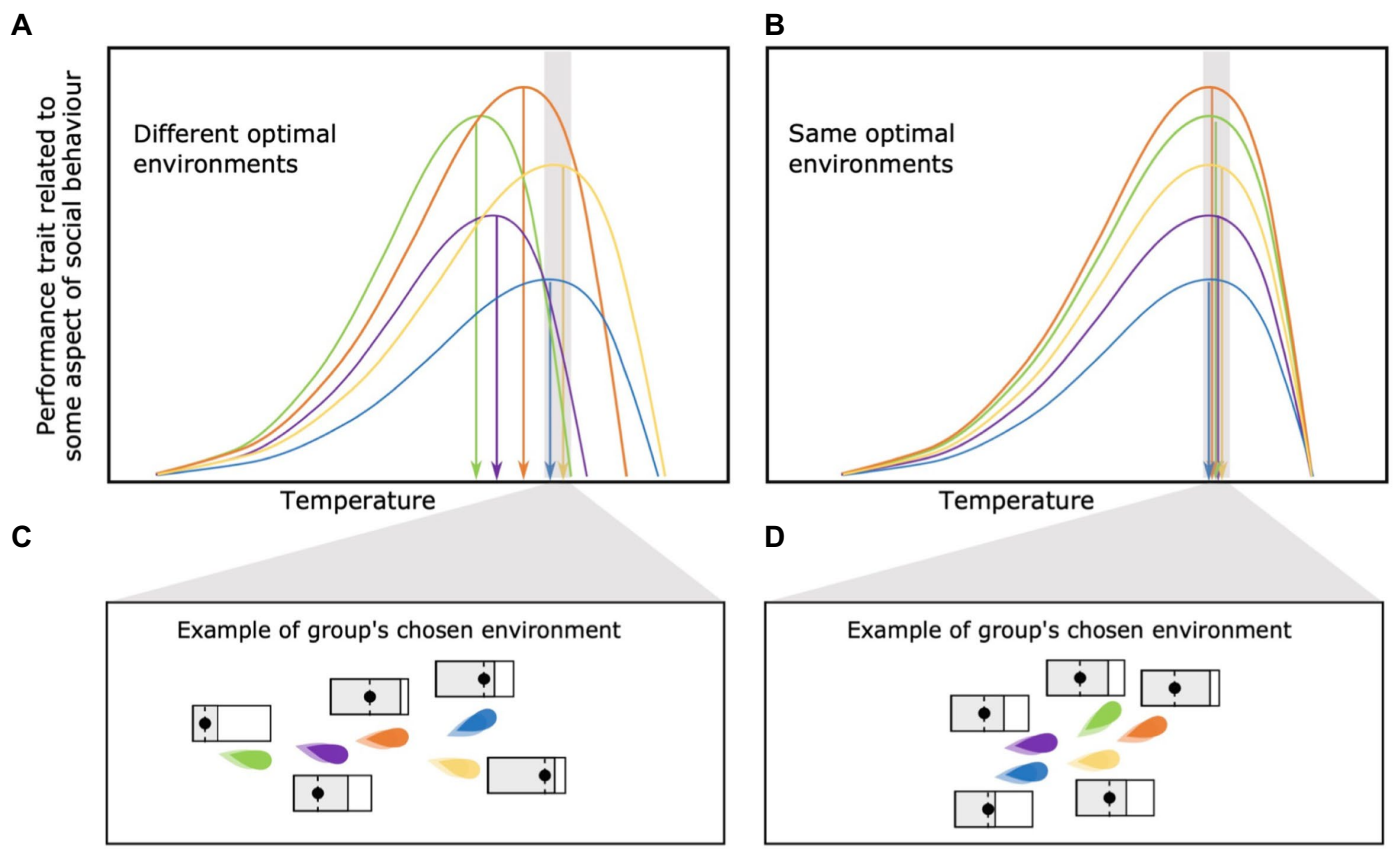
**(A)** Among-individual differences in optimal environments vs. **(B)** equal optimal environment among individuals belonging the same social group. One of the consequences of among-individual differences in optimal environments is that individuals may fit into different social “niches,” each with a different behavioral capacity and expression, depending on the physiological constraints they end up facing within the group’s chosen environment **(C)**. On the other hand, an similar optimal environments may lead to behavioral conformity among individuals **(D)**.

Different individuals within a social group are likely to have different environmental conditions at which their individual performance is optimized (green and blue lines in Figure 5A). It is also possible that the environmental conditions selected by the individual (or the group as a whole) may have nothing to do with optimizing their performance within a social group. For example, a group may choose to occupy a given location based solely on the availability of food or some other resource. In that case, the environmental conditions present at that point in space and time will determine how close each individual is operating relative to their individually optimal conditions and maximum capacity (Figures 5A,B). While individuals may be able to acclimate to environmental conditions, differences in rate of acclimation could still lead to very different trait values among individuals within the same environment. One possible consequence is that individuals may fit into vastly different social niches depending on the physiological constraints they end up facing within the (local) environment of the social group.

### Among-Individual Differences in Peak Performance Regardless of Environmental Optima

**Figure 6:**
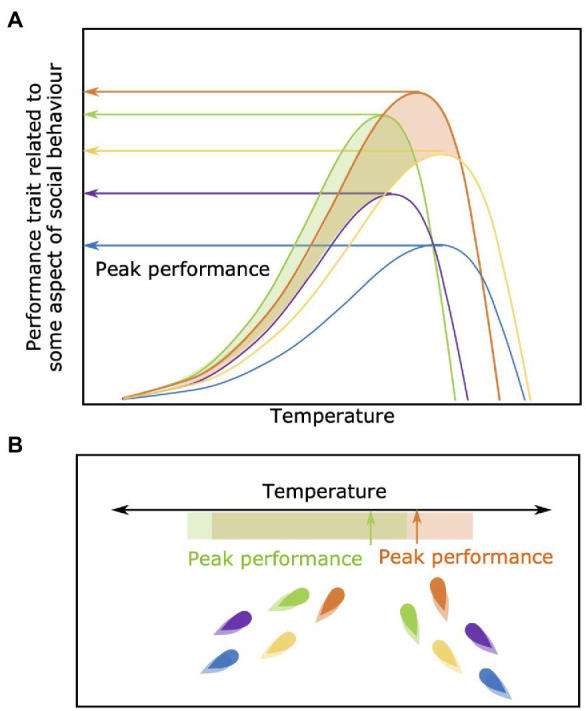
Among-individual differences in peak performance regardless of optima. In panel **(A)** individuals show variation in their absolute performance capacity across temperatures. In panel **(B)** individuals green and orange have a higher peak performance compared to the other individuals within the group, regardless of the optimal temperature for each. This elevated peak allows these two individuals to have a higher relative capacity for performance even if they are deviating from their own specific optimal temperature.

Even if measured at their optimum environmental conditions, individual group members will show different absolute peak levels of performance (orange and purple lines in Figure 6). Individuals are likely to try and take advantage of an increased performance potential and consequently influence their behavior and decision making within the context of the group. For example, an individual may choose to occupy a microhabitat within their group that brings that individual closer to its own peak performance capacity, or direct group movements to areas where that individual will derive an advantage due the local environmental conditions. For example, an individual that is relatively robust to variation in environmental oxygen availability (i.e., hypoxia; Killen et al., 2012b) could conceivably thrive socially in a moderately hypoxic environment if the competitive ability of its group-mates are reduced (although, the overall benefits of grouping for predator avoidance may decrease if overall group cohesion is impaired).

### Among-Individual Differences in Performance Breadth and Critical Limits

**Figure 7:**
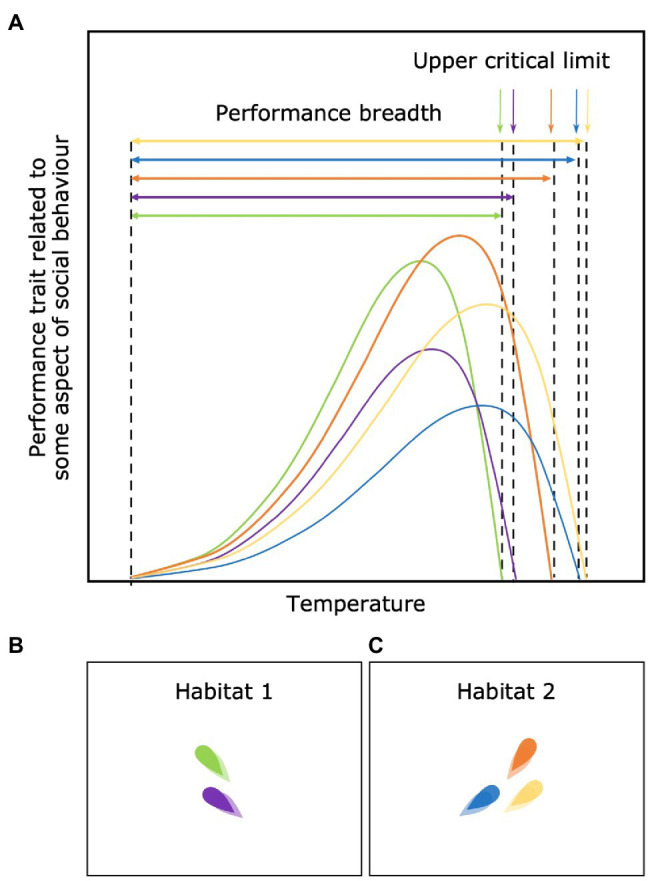
Among-individual differences in performance breadth and critical limits **(A)** and its consequences **(B,C)**. Variation in the breadth and critical thresholds limit the options of habitats available for each individual and promote among-group assortment [e.g. **(B,C)**].

Differences in performance curve shape may generate differences in the breadth over which individuals can function above particular thresholds of performance. For example, some individuals may be specialists (green individual in Figure 7A) and able to perform at a high level but only within a narrow environmental range, while others may be generalists (blue individual in Figure 7A) and able to perform over a wider range of environments but at a reduced absolute peak level of performance. The evidence for this trade-off between performance breadth and peak performance is however limited (Nati et al., 2016). There may also be among-individual differences in environmental tolerances of animals within a social group. Some individuals may simply be incapable of occupying the same environments as their conspecifics and even before this extreme point, have a sharper decline in performance (green and purple individual in Figure 7B). This variation in the breadth of environmental tolerance and critical thresholds for performance or survival should limit the habitats or environmental “options” available to groups with wide individual variation in such thresholds and may promote among-group assortment.

## Effects on Social Interactions

### Within-Group Competition and Conflict

Many social systems include dominance hierarchies, whereby individuals with greater resource holding potential have improved access to food, mates and/or other resources, and can often be found in locations within the group that reduce their risk of predation (Ward and Webster, 2016). Physiological traits are known to be important in the contests that establish dominance, as they correlate with competitive ability and also constrain the frequency, duration and intensity of contests, due to the build-up of lactic acid, for example, which limits anaerobic capacity (Briffa and Sneddon, 2007). A higher dominance status in contests has been shown to be associated with higher heart rate (Turbill et al., 2013), metabolic rate (Mccarthy, 2001), and aerobic scope (Killen et al., 2014). In turn, environmental variables can affect aggressive interactions *via* effects on physiology. For example, in cooler water, the cichlid fish *Cichlasoma paranaense* reduces aggressive interactions (Brandão et al., 2018), and the duration of fights between shore crabs (*Carcinus maenas*) is reduced in hypoxic conditions, associated with a greater accumulation of lactic acid during fights in hypoxia (Sneddon et al., 1998). Individuals experiencing cooler temperatures can compensate for reduced locomotor performance, however, through elevated aggression and be just as likely to win contests, as demonstrated in velvet geckos (*Oedura lesueurii*; Kondo and Downes, 2007).

Although these previous studies have shown that environmental variables can affect average levels of antagonistic interactions, variation in performance curves suggests that differences between individuals in resource holding potential and other forms of competitive ability (e.g., the ability to detect food sooner than others) is plastic, being dependent on the prevailing environmental conditions. This may mean, for example, that under some environmental conditions, individuals are more closely matched in fighting ability, which tends to result in more frequent, longer, and more intense contests (Schmitz and Baldassarre, 1992; Hack et al., 1997). Under other environmental conditions, differences in competitive ability between individuals may be magnified, resulting in clear winners, where contests are infrequent and easily won before they escalate. In cases where environmental changes over time are large enough to alter the rank order of physiological performance that determines dominance status, aggression may be more frequent and the dominance hierarchy less stable, which may explain changes in hierarchy stability with temperature (Kochhann et al., 2015). Changes in dominance may also be delayed or may not occur at all if there are carryover effects whereby a dominant individual is more likely to stay dominant (Hock and Huber, 2009), even if the environmental conditions become less favorable for its own phenotype. For example, established social rank can weaken the importance of physiological performance for competitive interactions in some species (Miln et al., 2021).

Even without dominance hierarchies and aggression, individuals within groups also consistently differ in their ability to exploit resources, such as food, during scramble competition (David et al., 2011). These individual differences can be explained by physiological processes that affect motivation for the resource, sensory perception of the resource and locomotory performance to acquire the resource before competitors (Miln et al., 2021). Differences among individuals in physiological performance curves will affect how resources are distributed within groups depending on the current environmental conditions. In environments where the variability in physiological performance of individuals is reduced, the distribution of resources to individuals will be more even, and the opposite effect is expected when environmental conditions magnify inter-individual variation within groups in physiological performance. If environmental conditions are varying at an appropriate scale over time, changes in the physiological performance rank of individuals may mean that average levels of resource use over time are more similar within the group than at any single time point. It is worth noting that under natural conditions, the availability of some resources is also dependent on environmental conditions. For example, scramble competition increases in colobus monkeys (*Colobus vellerosus*) within groups during the wet season when preferred foods are scarce (Teichroeb and Sicotte, 2018). Thus, the abundance of a resource over an environmental gradient must be considered alongside the physiological performances over that gradient. If, for example, the variability between individuals in performance during scramble competition is greatest when resources are highly abundant and not limiting, we may not expect to see a relationship between food intake and physiological performance, which may instead be seen when variability between individuals is reduced, but resources are highly limited.

In groups without clear dominance hierarchies, more subtle forms of conflict can occur without obvious aggression or scramble competition. Groups often make decisions regarding when, where and how to move, which requires coordination to maintain cohesion of the group. Multiple sources of variation between individuals within groups, whether short-term and transient (Kerth et al., 2006) or long-term and consistent (Bevan et al., 2018), have the potential to result in conflict over these collective decisions that require consensus (Conradt, 2012). In contexts such as when behaviors should be performed, compromise can be reached; in others where behavioral decisions are mutually exclusive, such as where to travel to, compromise is not possible (Wade et al., 2020). In this latter case, the ‘consensus costs’ paid by individuals who do not get their preferred outcome should, on average, be higher than when compromise is possible (Conradt and Roper, 2009). If such consensus costs are too high relative to the benefit of remaining with the group, groups can split (Ioannou et al., 2015). As the extent of variation between individuals often determines the extent of conflicting preferences within groups, variation in physiological performance curves would mean that the degree of conflicting preferences will be sensitive to environmental conditions. When environmental conditions result in reduced variation between individuals in physiological performance, preferences should be similar and this reduced within-group conflict should result in fast decisions and more cohesive, coordinated groups. In contrast, if greater physiological differences result in conflicting preferences, decisions are predicted to be slower, and the group may change their decision more frequently or even split. For example, the speed of travel of a group can be determined by the physiological performance of the group members, and a consensus decision on that speed will be easier when preferred speeds, based on physiological performance capacities, are similar (Sankey et al., 2019). A potential outcome is that groups may be quicker to make consensus decisions in relatively harsh or extreme environments when performance capacity is limited or among-individual variation is constrained.

### Social Niches and Social Conformity

While performance curves typically represent the maximum capacity that an individual has for a given physiological performance metric, individuals do not always opt to perform at their maximum capacity. This is partly because individuals within groups may need to coordinate behavior by either conforming to the group average or matching the behavior of a particularly influential individual (Brown and Irving, 2014; McCune et al., 2018). Alternatively, competition within groups can cause initial individual heterogeneity among group members to become amplified over time due to character displacement (the “social niche hypothesis”; Bergmüller and Taborsky, 2010; Montiglio et al., 2013, Jolles et al., 2020a). Previous research has attempted to determine whether conformity or the social niche hypothesis is a larger driver of behavior within social groups (Munson et al., 2021), however, changes in the environmental context can either constrain or expose phenotypic variation such that behavioral conformity or differentiation within a group is more or less possible in different environments. For example, behaviors may appear to conform if interindividual variation in performance curves is low and there are limited differences in potential performance. Alternatively, social niche formation should be optimized in environments where the differences in performance curves are the highest because there are the greatest initial differences in individual capacity for behavior.

Social dynamics may influence behavior to such an extent that individuals do not perform at their optimum across environmental contexts *via* behavioral conformity and the formation of social niches. This could have important feedbacks on differences in responses to changing environments despite individual performance curves. Even as the environment changes, individuals may be constrained by social dynamics to behaving similarly (or dissimilarly) from other group members, the predicted changes in performance based on individual performance curves may not be evident. For example, if fish conform to slower individuals in a group that also do not change as rapidly in their swim speed in response to changes in the environment, then the whole group will be limited in how much they respond to changes in the environment. Similarly, behavioral conformity and social niche formation should limit acclimation to environmental change within an individual. Even if an individual’s potential performance in one environmental context changes over time, they may not change their behavior if they are constrained to behaving similarly (or dissimilarly) from group members. Experiments that test performance curves for individuals alone and in groups would help to elucidate the influence of the group on individual performance across changing environments.

### Among and Within-Group Assortment

Animal groups are generally not randomly composed in nature, with individuals tending to assort according to various characteristics including body size, sex, age, or morphology (Krause et al., 2000; Jolles et al., 2020a). Animals both assort at the among-group level, with different phenotypes occurring in different groups, and the within-group level, with individuals occupying different spatial locations according to their phenotype and/or non-randomly interacting with similar individuals within the group. Furthermore, animals assort both actively, with individuals selecting which individuals they associate with, or passively, with individuals exhibiting spatiotemporal overlap due to shared habitat selection or attraction to a resource (Killen et al., 2017b). The potential influence of individual metabolic traits and locomotor capacity on among- and within-group assortment have been discussed in depth elsewhere (Killen et al., 2017b), sometimes in relation to sex-based differences in physiology and associated locomotor capacity and habitat preferences (Conradt and Roper, 2000; Ruckstuhl and Neuhaus, 2006), but there are a range of circumstances where the performance curves in particular could play an important role in these processes.

As environmental conditions change, differences in individual performance curves could lead to an increase or decrease in within-group variation in performance capacity. For example, environmental conditions may increase group movement speed and thereby lead to more within-group spatial assortment, such as slower individuals occupying posterior positions within the group. This has been observed in fish schools, in which the flow of water increasingly leads to individuals with lower aerobic scope to occupy positions in the back of the group (Killen et al., 2012a). Such effects could be further amplified or reduced depending on interactions among multiple environmental factors, such as that faster flowing water may carry more oxygen, which may thereby partly reduce assortment effects caused by the increased water flow rate. In contrast, an increase in water temperature may generate increased variation in locomotor capacity among group members and thereby enhance such assortment effects. In environments that produce greater amounts of variation among individuals within groups, groups may even split according to performance capacity, essentially leading to among-group assortment based on individual sensitivities to a particular environmental variable.

Among individual differences in environmental optima, tolerance breadths, or habitat preferences may also cause among-group assortment. For example, individual sensitivity to hypoxia stemming from performance curves may dictate which individuals occupy specific habitats or depths in aquatic environments (Joyce et al., 2016), and thus which conspecifics are available for them to interact with socially. Differences in energy requirements due to performance curves may also cause individuals to select different habitats and therefore spatially segregate (Michelangeli et al., 2018). Among-individual variation in changes in maintenance or active metabolism at different temperatures could cause individuals with a lower energy demand to select safer habitats, even if it means less access to food. Individuals with steeper increase in energy demand in response to temperature, however, may choose riskier habitats if it grants them increased access to food, and thereby group with individuals with a similar physiological and behavioral phenotype.

### Leader/Follower Dynamics

Choices in social group behavior (e.g., movement or a feeding event) can be reached by egalitarianism where all individuals reach consensus, or can be initiated by one or few individuals (i.e., leaders; Conradt and Roper, 2009). Leaders are only successful if followed by other group members, instigated voluntarily or as a result of hierarchical influence or dominance. Leaders in these groups often have better access to resources and make decisions for the group which may be at cost to others (King et al., 2008; although see McComb et al., 2001). In self-organized moving groups, leadership has been shown to propagate from the front of the group (Bumann and Krause, 1993; Nagy et al., 2010). Front positions are thought to be occupied by individuals who have more information about the surrounding environment or a greater need for resources and motivation to locate preferable environments (Ioannou et al., 2015), therefore leadership can depend on resource requirements linked to body size and sex (Fischhoff et al., 2007; Bierbach et al., 2020). The group members that successfully lead others and achieve their preferred outcome may be those with the highest physiological performance, for example those with the greatest aerobic capacity (Killen et al., 2012a) who can sustain more energetically-demanding positions or be better able to escape from attacks by predators, both costs of leadership associated with being at the front of moving groups (Ioannou et al., 2019). The ability to lead through spatial position or behavioral signaling could thus be constrained by physiological capacity, governed by an individual’s performance curve. However, the optimal leader may differ across environmental conditions. For example, it has been proposed that under benign conditions, individuals with the lowest metabolic rate and aerobic scope may become leaders as a way for the group to maintain high levels of cohesion, whereas under environmental stress individuals with a higher performance capacity take on leadership roles (Ward et al., 2018).

What is particularly interesting when considering group movement and physiological performance curves is that group movement may result in substantial changes to the environment that individuals experience. Those with greater influence on group movement may lead the group to locations with environmental conditions that improves (either absolutely or relatively to others in the group) their physiological performance, which may reinforce their position as leader. On the other hand, leaders’ preferred locations may be driven by factors other than their physiological performance, and due to inter-individual variation in physiological performance curves, a changed environment may shift which individual is most physiologically capable to lead subsequent group decisions. If groups are moving between locations which vary considerably in environmental parameters, individuals with narrower environmental tolerances may have the greatest motivation to lead, as they are likely to experience greater consensus costs if collective decisions take the group into locations of unpreferred environmental conditions. Additionally, other group members with wider tolerances may be less affected by environmental conditions, and may have less motivation to lead the group, despite potentially having a higher peak performance in changing environments. As the group encounters a less optimal environmental gradient then a leader’s capacity to lead may decrease due to variation in environmental tolerance. Moreover, if individual capacity to lead changes with performance curves, individuals may be more influential in different environments and could cause a switch in leadership from one individual to another. Alternatively, multiple individuals with similar performance curves could have the capacity to lead when experiencing a change in environment, causing a disruption to hierarchy and may lead to group splitting if the cost to staying with a group is too large (Ioannou et al., 2015).

### Collective Dynamics

Collective patterns, including the speed, alignment, synchronization, and movement tendency of animal groups, emerge *via* self-organizing mechanisms from the behavior and interactions of the individual group members (Couzin et al., 2002; Couzin and Krause, 2003, 2002). Hence, the phenotypic composition of groups, including the average behavior of and heterogeneity among group members, and its change over time, may strongly impact on collective dynamics (del Mar Delgado et al., 2018; Jolles et al., 2020a). Furthermore, changes in individual behavior and the interactions among grouping individuals in response to their environment coincides with changes in group-level patterns (Schaerf et al., 2017). Both the movement speed and social responsiveness of individuals are strongly linked to a range of physiological characteristics that may change depending on the environment, and thereby impact collective dynamics. For example, at higher temperatures, ectothermic animals may have less aerobic scope available, reducing their optimal and preferred movement speed and in turn result in slower, but potentially more cohesive groups. Alternatively, temperatures colder than optimal may also increase cohesion if overall activity is reduced *via* effects on individual performance curves (Bartolini et al., 2015). Similarly, changes in oxygen availability may differently impact the muscular functioning of individuals and, by changes in movement speed, impact collective dynamics.

Importantly, if individuals are far from their performance optimum, this could negatively impact their social responsiveness as they may be less able to and/or motivated to cognitively focus on their group mates. If environmental conditions push groups further from their physiological optima, this could then result in less synchronized groups and potentially cause groups to break apart. In a similar way, differences in metabolic requirements may, across changing resource availability in the environment, cause relative changes in individuals’ focus on goal-oriented vs. socially-oriented movements (i.e., motivation to stay together) and thereby impact the cohesion, speed, and alignment of groups. In many cases, social responsiveness is affected by sensory input, such as the extent to which individuals can see each other, and conditions such as increased water turbidity or habitat complexity will require individuals to slow down and be more socially responsive to not break social contact. This in turn may actually provide more scope for individuals with different physiological optima or different breadths of performance curves to stay together. Finally, the limits of group members’ physiological performance curves (or environmental tolerances) will determine how well they will be able to stay together and move across increasingly extreme conditions, as individuals may simply differ in the upper limits they can survive, such as in refuge pools of streams during extreme droughts.

## Effects on the Costs and Benefits of Grouping

### Social Foraging

Individuals in groups can benefit by increased access to food sources and the potential to exploit food resources discovered by others, but grouping can also result in competition (Ranta et al., 1993). As discussed earlier when considering within-group conflict, differences in physiological performance can allow some individuals to have disproportionately greater access to food. When physiological performance curves differ between individuals, the variability in how food is distributed between individuals should be driven by variation in physiological performance under the current environmental conditions. This could favor less competitively able individuals to actively leave groups, and the reduction in group size to potentially impact foraging efficiency and anti-predator benefits experienced by those group members that remain (Krause and Ruxton, 2002).

Predicting the role of physiological performance curves on social foraging may be dependent on the feedback between individuals’ physiological performance and changes in physiological state that occur during foraging. If the intake of food and time to satiation differs between individuals (Gifford et al., 2014; MacGregor et al., 2021), which could be determined by differences in physiological performance in the current environment, there may be conflict in the optimal time to stop foraging at that patch. If those with higher physiological performance have both faster food intake and greater influence over group decisions, then other individuals in the group will be less likely to forage for an adequate duration. This may act as a positive feedback which magnifies differences in physiological performance between individuals over the longer term. Because of variation in physiological performance curves, such a feedback would however be suppressed if foraging occurs under variable environmental conditions, favoring food intake of different individuals at different times.

The metabolic cost of digestion (Norin and Clark, 2017), which can impact physiological traits such as locomotion (Dupont-Prinet et al., 2009), may alter the spatial distribution of individuals within groups and their behavior during social foraging. For example, in common minnows (*Phoxinus phoxinus*) there are consistent among-individual differences in the time spent at the front of a shoal, with some fish spending more time in front than others and individuals in front tending to ingest most food items (McLean et al., 2018). After feeding however, individuals at the front move toward the back of the shoal, as a result of the reduction in aerobic metabolic scope available due to digestion (McLean et al., 2018). Satiated individuals may also reduce foraging and increase anti-predator vigilance to the benefit of others in the group (Arbon et al., 2020), dampening differences between individuals in food intake. Thus, both changing environmental conditions and inter-individual variation in physiological performance curves have potential to disrupt positive and negative feedback and thereby result in either a reduction or strengthening of inter-individual variation in food intake.

Feedbacks among physiological performance, environmental conditions and social behavior can be informed by recent research exploring how individual differences based on state can drive behavior, and how behavior can in turn drive differences in state (i.e., state-behavior feedbacks; Sih et al., 2015). Experimental tests with sticklebacks (*Gasterosteus aculeatus*) support the existence of feedbacks between risk-taking behavior and satiation, but even in this relatively simple case, these studies show that these feedbacks are unpredictable, without strong evidence in favor of negative or positive feedbacks (MacGregor et al., 2021). This suggests that integrating feedbacks into the interaction between physiological performance curves and social foraging will be challenging. Simulation modelling based on assumptions and parameters that are empirically determined may thus be an essential tool in this endeavor.

While there is strong evidence that group living improves rates of finding and exploiting food sources (Cvikel et al., 2015; Ioannou, 2017), if an individual’s success during collective foraging is related to their physiological performance, then performance these curves are likely to impact group-level performance when groups foraging in different environments or microhabitats. If groups are reliant on a small proportion of individuals to lead, for example those with information regarding the presence and location of food (Ioannou et al., 2015), and the ability of these individuals to lead is positively associated with their physiological (e.g., locomotory) performance, group foraging success will be greatest when environmental conditions are optimal for leading individuals. In contrast, if foraging is dependent on pooling information from many individuals in the group, such as in many eusocial insect colonies (Detrain and Deneubourg, 2009), then environmental conditions which favor the greatest average physiological performance may maximize foraging success. The environmental conditions that optimize group performance in foraging may thus be dependent on whether influence on foraging performance is distributed between many individuals or a few.

### Predator Avoidance

Reduced predation risk has been proposed as one of the main drivers for why most animals live in social groups (Krause and Ruxton, 2002). Importantly, the environmental context may alter predation risk for grouping animals, both by affecting predator behavior (Grigaltchik et al., 2012) as well as effects on group behavior. For example, if in a particular environment, phenotypic variance is high due to among-individual variation in performance curves, this may result in less cohesive groups, potentially reducing the anti-predator benefits for those individuals (Sogard and Olla, 1997). Groups that are more cohesive with less phenotypic variance benefit from the confusion effect whereby visual predators have reduced targeting accuracy when prey are phenotypically homogenous (Jeschke and Tollrian, 2007). Because of this, phenotypically different individuals can experience increased risk of predation relative to their group mates (the oddity effect; Theodorakis, 1989). As individual behavior and group composition are important aspects of predator avoidance (Farine et al., 2015; Blake et al., 2018), this suggests that not only should groups differ in their anti-predator success across environments as performance curves converge and diverge, but that individuals may prefer different groups as environments change. Different individuals are affected by the oddity effect to different extents (Rodgers et al., 2015). For example, an individual with particularly high-performance capacity in a given environment may be less susceptible to predation than an individual who has a low performance capacity relative to its groupmates, especially if these differences in physiological capacity manifest in behavioral differences (e.g., activity level) that make them more of less obvious to predators. Thus, as environments change, there may be differences in group membership, as individuals opt to forego or receive the full anti-predator benefits of being in a group. Additionally, there may be important ramifications on group level success if group predator avoidance is influenced by a leader, and if the identity or influence of a leader changes across an environmental gradient due to variation in performance curves.

### Social Learning and the Spread of Information

Many animals rely on social learning as a shortcut for behaviors linked to predation avoidance, migration, foraging, and reproduction (Brown and Laland, 2003; Mueller et al., 2013). The efficiency and benefits of social learning may change across an environmental gradient because of changes in the transmission of information from demonstrators, and perception and processing of information from learners. Information is mainly transmitted *via* sensory signals (cues), perceived, and transduced *via* sensory organs and processed *via* neurological pathways. Variation in the transmission, perception and processing of information may arise from alteration of the sensory signals themselves, which may be disrupted directly by changes in the environment, such as acoustic cues masked by human noise pollution (Radford et al., 2014), or visual cues reduced by increased water turbidity (Nieman and Gray, 2019). Physiological changes across environments can also impact the perception and processing of cues, as well as indirectly by changes in group cohesion and coordination, which will influence how well information will spread within groups (MacGregor et al., 2020).

Although in extreme environments sensory organs may even be directly damaged, less dramatic changes may occur in response to environmental changes that lead to physiological effects and impact individual signaling and perception. An example is hormonal disruptions such as modification of melatonin rhythms in birds with variation in night lighting (Dominoni et al., 2020). Neural transmission, brain functioning, and cognition may also vary across an environmental gradient with impacts on social learning capacities. A well-known example is honey bees exposed to pesticides, which have reduced brain functioning (Klein et al., 2017) that may translate into a weaker ability to learn how to localize food from waggle dances (von Frisch, 1967). As with the development of social niches and leader and follower behaviors, greater within-group variation in individuals’ physiological performance should favor more distinct demonstrator and learner roles, which can result in conflict over preferred group dynamics (MacGregor et al., 2020). Furthermore, variation in rank order across environments, such as a change in rank order of performance capacity at higher temperature (Figure 2), may result in a change in which individuals are demonstrators and which are learners. If relative changes in physiological performance and preferences promote a less stable group composition, reduced familiarity with the demonstrator and other individuals belonging to the group may affect the social transmission of information (Hasenjager and Dugatkin, 2017; Barrett et al., 2019).

Group-level behaviors and dynamics are likely to vary across environments (e.g., increased water temperature and hypoxia may decrease group cohesion in aquatic ectotherms), which can strongly affect how social information is transmitted (e.g., visual information, MacGregor et al., 2020). Any changes in group cohesion could in turn alter the potential for information transfer among groupmates due to changes in spatial distances among individuals and their ability to give and receive social cues (Pineda et al., 2020). In addition, the extent that individuals use social learning can be dependent on group behavioral composition. For example, using network-based diffusion analysis it has been found that, in guppies, social learning rate is higher in both bold and risk averse individuals when they are part of groups dominated by risk-averse individuals or mixed groups and there is a bold demonstrator (Hasenjager et al., 2020). Across gradients of environmental variation, among- and within-individual differences in behavioral expression in relation to performance curves may therefore lead to variation in social learning. If, across such gradients, the risks and benefits associated with social learning change (e.g., different reliability and efficiency of the transmission and perception of information within groups), non-optimal environments may lead to changes in social learning (e.g., I’Anson Price et al., 2019).

### Disease and Parasite Transfer

Disease transfer and parasite load can both be affected by the environmental context (Aeby and Santavy, 2006) and social behavior of animals (Hawley et al., 2011). Social behavior can increase risk of disease and parasite transfer between individuals (Ezenwa, 2004), especially when groups are more cohesive because of the closer proximity between individuals (Bull et al., 2012). This is detrimental because parasites can have both direct costs to infected individuals and indirect costs to group members (Granroth-Wilding et al., 2015). Indeed, avoidance of disease and parasite transfer has been proposed as one of the key factors keeping group sizes small in some species (Alexander, 1974; Huffman and Chapman, 2009; Patterson and Ruckstuhl, 2013), although “socially transferred” infection resistance can in certain cases improve immune abilities in group dwelling organisms (Traniello et al., 2002; Ugelvig and Cremer, 2007).

However, the relationship between social behavior and disease transfer may be influenced by individual differences in performance curves. For example, if group cohesion is altered due to changes in phenotypic variance in performance curves, rates of disease and parasite transfer could also change. Furthermore, if shifting environmental conditions affect optimal group membership due to altered physiological performance and individuals move between groups, this could increase disease transfer between groups. Previous work suggests that increased space use relates to parasite load (Boyer et al., 2010) and that this can be influenced by the environmental context (Spiegel et al., 2015). If environmental conditions change rapidly, this could cause decreased group stability and more rapid transfer of individuals (and their diseases) between groups as individuals spread out.

Additionally, infection may alter an individuals’ behavior such that it becomes less social. This could be for a variety of reasons including active avoidance by healthy individuals (Poirotte et al., 2017), a response to reduce disease transmission and increase inclusive fitness (Heinze and Walter, 2010), or manipulation by the infecting agent (Hughes et al., 2004). However, an apparent decrease in sociability could also occur because the physiological costs of disease make maintaining group membership challenging. Importantly, susceptibility to disease can change across environmental gradients, both because of differences in parasite performance curves (Sheets et al., 2021) and because of changes in the potential host’s immune function (Adamo and Lovett, 2011; Makrinos and Bowden, 2016). Further, differences in individual performance curves may mean that individuals are differentially susceptible to disease or parasite infection (Kurtz et al., 2000), which could relate to position within a social hierarchy or leader-follower dynamics (Larcombe et al., 2013; Snyder-Mackler et al., 2016). Different individuals may thus be more susceptible to disease or parasite transmission across an environmental gradient which could influence their social behavior and potentially group dynamics, particularly if susceptible individuals are leaders. Finally, impairments caused by diseases or parasites can reduce individual speed and mobility, which in turn may influence their ability and motivation to be social (Jolles et al., 2020c). There may therefore be synergistic effects on social behavior between disease or parasitic infection and other factors that affect locomotor performance, such as temperature or hypoxia.

### Migrations and Range Expansions

Group movement occurs at different spatial and temporal scales. At small scales, within a population’s distribution, group movement is generally driven by organisms’ motivation and necessity to find resources or shelter. Such movements, from one resource patch to another or from one tree to the other for cover, often relies on social interactions where the presence of more experienced individuals or with knowledge for specific information such as the location of food resources can guide naïve individuals or transmit the information to the other group members (Mueller et al., 2013; Berdahl et al., 2018). At a larger scale, movements are associated with migration or range expansion (Cote et al., 2017) and social interactions still have a central role. Indeed social interactions can improve the accuracy of group navigation (Simons, 2004; Berdahl et al., 2018) and reduces energy expenditures (Herskin and Steffensen, 1998; Marras et al., 2015). However, despite numerous advantages there are also potential costs to individuals associated with group movement, including coordination (Nagy et al., 2010) and consensus costs (Conradt and Roper, 2009) such as adjustment of individual performance to match the group performance and individual differences in lower or upper limits of physiological performance across environmental gradients (Figures 1, 7). Therefore, as groups move across various spatial scales and environments, environmental effects on performance curves will continuously modulate group functioning and performance of individuals within the group.

One response of organisms to unsuitable environmental conditions is to relocate into more favorable habitats. However, relocation is strictly linked to movement behavior including group movement and to the ability to settle. If individual variation in performance curves affects group movement then reduced relocation opportunities may be expected under certain environments. For example, during drought, especially in mediterranean climates, parts of rivers dry up completely, requiring individuals from fish populations in those rivers to move to deeper safe refuges that do not dry up. In those conditions individual physiological and behavioral traits may be essential for group movement (see Box 1 for more details). However, not all individuals perform equally well in new environments and even if large scale movements occur, they may come at the cost of group re-arrangement.

## Experimental Approaches

While gaining a better understanding of the relationships between performance curves and social behavior is critically important in a changing world, these are not easy relationships to decipher. Ideally, we need performance data for individuals tested repeatedly across an environmental gradient and then in groups across the same range. Acquiring detailed data to be able to construct individual performance curves requires many repeated measures of the same individuals across a range of conditions of the same environmental variable. Accurate and precise estimates of individual variation in a reaction norm require relatively large sample sizes and each individual tested multiple times (Martin et al., 2011; van de Pol, 2012; Allegue et al., 2017). Estimating performance curves can be even more sample intensive, particularly because the important variation is typically greater in estimating higher order parameters associated with curve shape than for those associated with offset or slope (Murren et al., 2014). To then consider the social axis as we discuss here, the number of individuals required for a study will be even larger.

Still, these studies are possible, particularly with the advent of automated techniques and low cost open source electronics (Jolles, 2021). Experiments with social groups that directly examine the influence of food availability and predator presence across environmental gradients may help address these issues, as will validation of detailed patterns seen in lab studies with less granular studies done in wild populations. Emerging technologies that allow high-resolution tracking of animals in the wild (Guzzo et al., 2018), combined with transmitters or loggers that acquire physiological data (e.g., heart rate; Williams et al., 2021) across habitats and environments will be particularly useful for allowing researchers to examine these questions. The general approach begins with measuring the same individuals repeatedly for a physiological trait and their behavior (e.g., locomotor capacity, temperature preference, spatial position) across a range of conditions (e.g., temperature, oxygen availability, turbidity) to construct individual performance curves. It is important to consider that, due to the large number of measurements required, not all traits can be easily investigated, especially those that are relatively invasive such as those relating to tissues or organ level physiological performance. Notedly, because lab studies often test animals when they are otherwise at relatively benign conditions, there have been recent calls to improve ecological relevance by confirming laboratory studies of performance curves with field data (Childress and Letcher, 2017). This may be particularly important when seeking to understand group behavior, the patterns of which are often the result of trade-offs between individual foraging needs and the benefits of groups for predator protection.

After repeatedly measuring individual performance curves in isolation, animals should be assigned to groups. The method for group assignment should be considered carefully depending on the exact question being asked. For example, if researchers are interested in how performance in a given environmental context affects group assortment, animals should be allowed to assort themselves. However, if the question relates more to how groups manage performance of different individuals as conditions change, group assignment can be done by the experimenter. This also requires careful consideration such as whether to optimize the performance of all individuals, the performance of the group as a whole or the differences between individuals.

Additionally, experimenters will need to decide whether they are going to measure the performance of a few focal individuals or all individuals in the social groupings. Due to the time and work involved in collecting performance curves on each additional animal, this is a serious consideration. While measuring every individual in a group provides more information, it can functionally limit the number of groups that can reasonably be measured. Whether fewer individuals per group can be measured depends on the exact question being asked. Importantly, even if the ultimate question relates to individual performance, it may be important to construct performance curves for all individuals in a group if the question focuses on how the individual relates to group performance and whether the important metric is average group performance or individual rank. While this type of experiment can be time intensive, without a better understanding of how individual performance curves influence social behavior traits and group performance, we will be unable to adequately predict how animal groups respond to changing environmental conditions.

BOX 1 **Methodological Case Study: Using performance curves and social dynamics to understand how fish deal with droughts**

Many freshwater ecosystems are characterized by natural seasonal fluctuations of their water cycle, including droughts and floods (Lennox et al., 2019). Despite being an integral part of the ecosystem, droughts have strong impacts on fish and other aquatic biota by increases in water temperature, deoxygenation, and reducing habitat availability and connectivity by reductions in water flow (Magoulick and Kobza, 2003; Mas-Martí et al., 2010). In fluvial systems in particular, severe droughts can result in complete sections of rivers to dry up, confining fish to few refugia with very extreme abiotic conditions, intense competition, and high predation risk (Magoulick and Kobza, 2003). Physiological performance curves are likely to directly affect how individual fish cope with these strong environmental changes, but also indirectly through various social effects, whereby the responses and capabilities of individual animals to drought may be compromised or enhanced, influenced by the phenotypic composition of groups (see main text; Killen et al., 2017b; Jolles et al., 2020a). For example, fish more sensitive to temperature increases may be the first to leave areas that may dry up later and thereby could act as leaders that “rescue” individuals with broader performance curves and correspondingly wider thermal tolerances. It is also possible that, in pools with low oxygen availability and warm water, competitive interactions change considerably relative to non-drought conditions, putting individuals with narrower performance curves (e.g., in terms of aerobic scope) at risk.

To better understand the above types of scenarios in terms of how fish may deal with the severe effects of droughts, we first need to understand how individual fish cope with changes in their environment related to drought at both the behavioral and physiologic levels. To start, one could decide to focus on hypoxia linked to drought and determine the physiological performance curves in terms of metabolic capacity and activity change across decreasing levels of dissolved oxygen the water. To do this, a replicated setup of 16 respirometry chambers could be used to measure the standard metabolic rate (SMR) and aerobic scope (AS) of fish during acute exposure to various levels of oxygen availability observed in the wild, e.g., 100, 75, 50, and 25% air saturation. Fish would be tested in a random order in terms of oxygen treatment to avoid temporal effects, and could be tested on alternative days such that two batches could be run on following days. In that way it would be possible to test 32 fish on all four treatment levels in approximately 8days.

Physiological experiments could be complemented with automated behavioral experiments to determine how fish behaviorally respond to different levels of oxygenation, particularly spontaneous activity, air-breathing, and potential escape (longer directed movement) behavior. For this, fish could be tested individually in arenas, filled with water at a specific oxygen level and containing rocks and partitions to provide structure. A system of replicated setups could be used with automated recording (e.g., pirecorder) and tracking of the fishes’ movements, such that all 32 fish could be tested on one treatment level per day (randomized).

After acquiring the individual measures, fish could be tested for social behavior in larger arenas in small groups of different compositions in terms of their physiological performance. A range of different questions could be investigated, each requiring a different type of homo- and heterogenization. To start, one could focus on understanding the effects of individuals’ breadth of performance curve in terms of metabolic phenotype on competitiveness in a social foraging scenario. Thereby groups, such as with a group size of 6 fish, could be composed of individuals with small and large performance breadths and exposed to an open arena with hidden foraging patches and repeatedly tested across the four oxygen treatment levels. Manual video observations will help determine the cumulative food intake of the individual fish with automated tracking linking this to changes in the individual movement and social interaction rules (see, e.g., Jolles et al., 2017; MacGregor et al., 2020). Additional experiments could be performed in which social trials are run at differing levels of hypoxia such that among-individual variation in performance capacity and behavior could be manipulated according to each individual’s performance curves, and the resulting effects on social behavior observed.

With careful planning of the physiological and behavioral measurements, while properly accounting for acclimatization and randomizing for order and treatment effects, it should be feasible, following the above, to get a sample size of 96 fish tested within 8weeks. In the foraging experiment described above, the dataset would have 384 unique individual scores in terms of SMR, AS, individual activity, and social activity to determine individual physiological performance curves and heterogeneity therein as well as the effects of this heterogeneity on group functioning in terms of social foraging (at the baseline foraging condition, presumably at normoxia). Note that this experimental design only considers acute exposures to the various levels of oxygen availability. A study could also start with fish acclimated (for at least 2weeks) to the various hypoxia treatments, but this would obviously increase the amount of time needed for the project if individual performance curves are to be constructed after acclimation and subsequent testing at each condition.

## Concluding Remarks

It is becoming increasingly clear that: (1) animal social behavior is linked with the physiological performance capacity of individuals; and (2) physiological performance is strongly influenced by environmental factors. Accordingly, it is apparent that a research approach that involves estimation of performance curves is required to fully understand how environmental factors influence social behavior. Conversely, the measurement of performance curves has been a central feature of the study of comparative physiology and ecophysiology during the last several decades, but in virtually all cases has only been applied to individual animals and devoid of any social context. As individual heterogeneity within groups is a known driver of leadership, conflict, cohesion and coordination, environmental effects on phenotypic variation should ultimately influence behaviors at the group level. As wild animals are being exposed to increasing environmental changes, an integration of physiological performance curves with the measurement of social behavior will be key for understanding how such changes affect group living and associated ecological phenomena. We therefore encourage increased collaboration among ecophysiologists and researchers that investigate animal social behavior to achieve a more complete understanding of how species will respond to environmental change.

## Data Availability Statement

The original contributions presented in the study are included in the article/supplementary material, further inquiries can be directed to the corresponding author.

## Author Contributions

SK and CI contributed to conception and design of the manuscript. DC, LC, JJ, and AM contributed to further idea development and refinement. SK coordinated manuscript writing and compiled manuscript drafts. All authors drafted specific sections of the manuscript. DC, SK, JJ, and CI designed and produced figures with additional input from LC and AM. All authors contributed to manuscript revision, read, and approved the submitted version.

## Conflict of Interest

The authors declare that the research was conducted in the absence of any commercial or financial relationships that could be construed as a potential conflict of interest.

## Publisher’s Note

All claims expressed in this article are solely those of the authors and do not necessarily represent those of their affiliated organizations, or those of the publisher, the editors and the reviewers. Any product that may be evaluated in this article, or claim that may be made by its manufacturer, is not guaranteed or endorsed by the publisher.

## References

[ref1] Adamo S. A. Lovett M. M. (2011). Some like it hot: the effects of climate change on reproduction, immune function and disease resistance in the cricket *Gryllus texensis*. J. Exp. Biol. 214, 1997–2004. doi: 10.1242/jeb.056531 21613515

[ref2] Aeby G. S. Santavy D. L. (2006). Factors affecting susceptibility of the coral Montastraea faveolata to black-band disease. Mar. Ecol. Prog. Ser. 318, 103–110. doi: 10.3354/meps318103

[ref3] Alexander R. D. (1974). The evolution of social behavior. Annu. Rev. Ecol. Syst. 5, 325–383. doi: 10.1146/annurev.es.05.110174.001545

[ref4] Allegue H. Araya-Ajoy Y. G. Dingemanse N. J. Dochtermann N. A. Garamszegi L. Z. Nakagawa S.. (2017). Statistical quantification of individual differences (SQuID): an educational and statistical tool for understanding multilevel phenotypic data in linear mixed models. Methods Ecol. Evol. 8, 257–267. doi: 10.1111/2041-210X.12659

[ref6] Arbon J. J. Kern J. M. Morris-Drake A. Radford A. N. (2020). Context-dependent contributions to sentinel behaviour: audience, satiation and danger effects. Anim. Behav. 165, 143–152. doi: 10.1016/j.anbehav.2020.04.021

[ref7] Armstrong J. D. Millidine K. J. Metcalfe N. B. (2011). Ecological consequences of variation in standard metabolism and dominance among salmon parr. Ecol. Freshw. Fish 20, 371–376. doi: 10.1111/j.1600-0633.2011.00486.x

[ref8] Barrett B. Zepeda E. Pollack L. Munson A. Sih A. (2019). Counter-culture: does social learning help or hinder adaptive response to human-induced rapid environmental change? Front. Ecol. Evol. 7:183. doi: 10.3389/fevo.2019.00183

[ref9] Barrionuevo W. R. Burggren W. W. (1999). O2 consumption and heart rate in developing zebrafish (Danio rerio): influence of temperature and ambient O2. Am. J. Phys. Regul. Integr. Comp. Phys. 276, R505–R513. doi: 10.1152/ajpregu.1999.276.2.R505 9950931

[ref10] Bartheld J. L. Artacho P. Bacigalupe L. (2017). Thermal performance curves under daily thermal fluctuation: a study in helmeted water toad tadpoles. J. Therm. Biol. 70, 80–85. doi: 10.1016/j.jtherbio.2017.09.008, PMID: 29108561

[ref11] Bartolini T. Butail S. Porfiri M. (2015). Temperature influences sociality and activity of freshwater fish. Environ. Biol. Fish 98, 825–832. doi: 10.1007/s10641-014-0318-8

[ref12] Bauer C. M. Skaff N. K. Bernard A. B. Trevino J. M. Ho J. M. Romero L. M.. (2013). Habitat type influences endocrine stress response in the degu (*Octodon degus*). Gen. Comp. Endocrinol. 186, 136–144. doi: 10.1016/j.ygcen.2013.02.036, PMID: 23518483

[ref13] Beauchamp G. (2004). Reduced flocking by birds on islands with relaxed predation. *Proceedings of the Royal Society of London*. Series B Biologic. Sci. 271, 1039–1042. doi: 10.1098/rspb.2004.2703, PMID: 15293857 PMC1691692

[ref14] Berdahl A. M. Kao A. B. Flack A. Westley P. A. H. Codling E. A. Couzin I. D.. (2018). Collective animal navigation and migratory culture: from theoretical models to empirical evidence. Philosophic. Trans. Royal Society B Biologic. Sci. 373:20170009. doi: 10.1098/rstb.2017.0009, PMID: 29581394 PMC5882979

[ref002] Bergmüller R. Taborsky M. (2010). Animal personality due to social niche specialisation. Trends Ecol. Evol. 25, 504–511. 20638151 10.1016/j.tree.2010.06.012

[ref15] Bevan P. A. Gosetto I. Jenkins E. R. Barnes I. Ioannou C. C. (2018). Regulation between personality traits: individual social tendencies modulate whether boldness and leadership are correlated. Proc. R. Soc. B Biol. Sci. 285:20180829. doi: 10.1098/rspb.2018.0829, PMID: 29899075 PMC6015863

[ref16] Bierbach D. Mönck H. J. Lukas J. Habedank M. Romanczuk P. Landgraf T.. (2020). Guppies prefer to follow large (robot) leaders irrespective of own size. Front. Bioeng. Biotechnol. 8:441. doi: 10.3389/fbioe.2020.00441 32500065 PMC7243707

[ref17] Blake C. A. Andersson M. L. Hulthén K. Nilsson P. A. Brönmark C. (2018). Conspecific boldness and predator species determine predation-risk consequences of prey personality. Behav. Ecol. Sociobiol. 72:133. doi: 10.1007/s00265-018-2544-0

[ref18] Boyer N. Réale D. Marmet J. Pisanu B. Chapuis J.-L. (2010). Personality, space use and tick load in an introduced population of Siberian chipmunks Tamias sibiricus. J. Anim. Ecol. 79, 538–547. doi: 10.1111/j.1365-2656.2010.01659.x 20202009

[ref19] Brandão M. L. Colognesi G. Bolognesi M. C. Costa-Ferreira R. S. Carvalho T. B. Gonçalves-de-Freitas E. (2018). Water temperature affects aggressive interactions in a Neotropical cichlid fish. Neotropic. Ichthyol. 16:e170081. doi: 10.1590/1982-0224-20170081

[ref20] Breves J. P. Specker J. L. (2005). Cortisol stress response of juvenile winter flounder (*Pseudopleuronectes americanus*, Walbaum) to predators. J. Exp. Mar. Biol. Ecol. 325, 1–7. doi: 10.1016/j.jembe.2005.04.019

[ref21] Briffa M. Sneddon L. U. (2007). Physiological constraints on contest behaviour. Funct. Ecol. 21, 627–637. doi: 10.1111/j.1365-2435.2006.01188.x

[ref22] Brijs J. Sandblom E. Rosengren M. Sundell K. Berg C. Axelsson M.. (2019). Prospects and pitfalls of using heart rate bio-loggers to assess the welfare of rainbow trout (*Oncorhynchus mykiss*) in aquaculture. Aquaculture 509, 188–197. doi: 10.1016/j.aquaculture.2019.05.007

[ref001] Brown C. Irving E. (2014). Individual personality traits influence group exploration in a feral guppy population. Behav. Ecol. 25, 95–101.

[ref23] Brown C. Laland K. N. (2003). Social learning in fishes: a review. Fish Fish. 4, 280–288. doi: 10.1046/j.1467-2979.2003.00122.x

[ref24] Bull C. M. Godfrey S. S. Gordon D. M. (2012). Social networks and the spread of salmonella in a sleepy lizard population. Mol. Ecol. 21, 4386–4392. doi: 10.1111/j.1365-294X.2012.05653.x, PMID: 22845647

[ref25] Bulté G. Blouin-Demers G. (2006). Cautionary notes on the descriptive analysis of performance curves in reptiles. J. Therm. Biol. 31, 287–291. doi: 10.1016/j.jtherbio.2005.11.030

[ref26] Bumann D. Krause J. (1993). Front individuals lead in shoals of three-spined sticklebacks (*Gasterosteus aculeatus*) and juvenile roach (*Rutilus rutilus*). Behaviour 125, 189–198. doi: 10.1163/156853993X00236

[ref27] Burton T. Killen S. S. Armstrong J. D. Metcalfe N. B. (2011). What causes intraspecific variation in resting metabolic rate and what are its ecological consequences? Proc. R. Soc. B Biol. Sci. 278, 3465–3473. doi: 10.1098/rspb.2011.1778, PMID: 21957133 PMC3189380

[ref28] Careau V. Biro P. A. Bonneaud C. Fokam E. B. Herrel A. (2014). Individual variation in thermal performance curves: swimming burst speed and jumping endurance in wild-caught tropical clawed frogs. Oecologia 175, 471–480. doi: 10.1007/s00442-014-2925-7, PMID: 24652528

[ref29] Chamberlain A. C. Ioannou C. C. (2019). Turbidity increases risk perception but constrains collective behaviour during foraging by fish shoals. Anim. Behav. 156, 129–138. doi: 10.1016/j.anbehav.2019.08.012

[ref30] Chan A. A. Y.-H. Giraldo-Perez P. Smith S. Blumstein D. T. (2010). Anthropogenic noise affects risk assessment and attention: the distracted prey hypothesis. Biol. Lett. 6, 458–461. doi: 10.1098/rsbl.2009.1081, PMID: 20164080 PMC2936217

[ref32] Childress E. S. Letcher B. H. (2017). Estimating thermal performance curves from repeated field observations. Ecology 98, 1377–1387. doi: 10.1002/ecy.1801, PMID: 28273358

[ref33] Conradt L. (2012). Models in animal collective decision-making: information uncertainty and conflicting preferences. Interface Focus 2, 226–240. doi: 10.1098/rsfs.2011.0090, PMID: 23565335 PMC3293206

[ref34] Conradt L. Roper T. J. (2000). Activity synchrony and social cohesion: a fission-fusion model. Proc. Royal Soc. London Series B Biologic. Sci. 267, 2213–2218. doi: 10.1098/rspb.2000.1271, PMID: 11413635 PMC1690793

[ref35] Conradt L. Roper T. J. (2009). Conflicts of interest and the evolution of decision sharing. Philosophic. Trans. Royal Soc. B Biologic. Sci. 364, 807–819. doi: 10.1098/rstb.2008.0257, PMID: 19073479 PMC2689720

[ref36] Cooper B. Adriaenssens B. Killen S. S. (2018). Individual variation in the compromise between social group membership and exposure to preferred temperatures. Proc. R. Soc. B Biol. Sci. 285:20180884. doi: 10.1098/rspb.2018.0884, PMID: 29899078 PMC6015869

[ref37] Cote J. Bocedi G. Debeffe L. Chudzińska M. E. Weigang H. C. Dytham C.. (2017). Behavioural synchronization of large-scale animal movements – disperse alone, but migrate together? Biol. Rev. 92, 1275–1296. doi: 10.1111/brv.12279, PMID: 27151681

[ref38] Couzin I. D. Krause J. James R. Ruxton G. D. Franks N. R. (2002). Collective memory and spatial sorting in animal groups. J. Theor. Biol. 218, 1–11. doi: 10.1006/jtbi.2002.3065, PMID: 12297066

[ref003] Couzin I. D. Krause J. (2003). Self-organization and collective behavior in vertebrates. Adv. Study Behav. 32, 10–1016.

[ref39] Currie H. A. L. White P. R. Leighton T. G. Kemp P. S. (2020). Group behavior and tolerance of Eurasian minnow (Phoxinus phoxinus) in response to tones of differing pulse repetition rate. J. Acoustical Soc. America 147, 1709–1718. doi: 10.1121/10.0000910, PMID: 32237844

[ref40] Cvikel N. Egert Berg K. Levin E. Hurme E. Borissov I. Boonman A.. (2015). Bats aggregate to improve prey search but might be impaired when their density becomes too high. Curr. Biol. 25, 206–211. doi: 10.1016/j.cub.2014.11.010, PMID: 25578909

[ref41] David M. Cézilly F. Giraldeau L.-A. (2011). Personality affects zebra finch feeding success in a producer–scrounger game. Anim. Behav. 82, 61–67. doi: 10.1016/j.anbehav.2011.03.025

[ref44] del Mar Delgado M. Miranda M. Alvarez S. J. Gurarie E. Fagan W. F. Penteriani V.. (2018). The importance of individual variation in the dynamics of animal collective movements. Philosophic. Trans. Royal Soc. B Biologic. Sci. 373:20170008. doi: 10.1098/rstb.2017.0008, PMID: 29581393 PMC5882978

[ref45] Detrain C. Deneubourg J. L. (2009). “Social cues and adaptive foraging strategies in ants,” in Food exploitation by social insects. eds. Jarau S. Hrncir M. (Boca Raton, FL: CRC Press), 29–54.

[ref46] Dingemanse N. J. Kazem A. J. Réale D. Wright J. (2010). Behavioural reaction norms: animal personality meets individual plasticity. Trends Ecol. Evol. 25, 81–89. doi: 10.1016/j.tree.2009.07.013 19748700

[ref47] Domenici P. Steffensen J. F. Marras S. (2017). The effect of hypoxia on fish schooling. Philosophic. Trans. Royal Soc. B Biologic. Sci. 372:20160236. doi: 10.1098/rstb.2016.0236, PMID: 28673914 PMC5498298

[ref48] Dominoni D. M. Halfwerk W. Baird E. Buxton R. T. Fernández-Juricic E. Fristrup K. M.. (2020). Why conservation biology can benefit from sensory ecology. Nat. Ecol. Evol. 4, 502–511. doi: 10.1038/s41559-020-1135-4 32203474

[ref49] Dupont-Prinet A. Claireaux G. McKenzie D. J. (2009). Effects of feeding and hypoxia on cardiac performance and gastrointestinal blood flow during critical speed swimming in the sea bass Dicentrarchus labrax. Comp. Biochem. Physiol. A Mol. Integr. Physiol. 154, 233–240. doi: 10.1016/j.cbpa.2009.06.015 19559805

[ref50] Ezenwa V. O. (2004). Host social behavior and parasitic infection: a multifactorial approach. Behav. Ecol. 15, 446–454. doi: 10.1093/beheco/arh028

[ref51] Farine D. R. Montiglio P.-O. Spiegel O. (2015). From individuals to groups and Back: The evolutionary implications of group phenotypic composition. Trends Ecol. Evol. 30, 609–621. doi: 10.1016/j.tree.2015.07.005, PMID: 26411618 PMC4594155

[ref52] Fischhoff I. R. Sundaresan S. R. Cordingley J. Larkin H. M. Sellier M.-J. J. Rubenstein D. I. (2007). Social relationships and reproductive state influence leadership roles in movements of plains zebra, Equus burchellii. Anim. Behav. 73, 825–831. doi: 10.1016/j.anbehav.2006.10.012

[ref53] Fisher D. N. Kilgour R. J. Siracusa E. R. Foote J. R. Hobson E. A. Montiglio P.-O.. (2021). Anticipated effects of abiotic environmental change on intraspecific social interactions. Biol. Rev. doi: 10.1111/brv.12772 [Ahead of preprint], PMID: 34212487

[ref54] Fry F. E. J. (1971). “The effect of environmental factors on the physiology of fish,” in Fish physiology, Environmental relations and behavior. *Vol.* 6. eds. Hoar W. S. Randall D. J. (New York: Academic Press), 1–98.

[ref55] Gifford M. E. Clay T. A. Careau V. (2014). Individual (co)variation in standard metabolic rate, feeding rate, and exploratory behavior in wild-caught semiaquatic salamanders. Physiol. Biochem. Zool. 87, 384–396. doi: 10.1086/675974, PMID: 24769703

[ref56] Gilbert A. L. Miles D. B. (2016). Food, temperature and endurance: effects of food deprivation on the thermal sensitivity of physiological performance. Funct. Ecol. 30, 1790–1799. doi: 10.1111/1365-2435.12658

[ref57] Gilbert A. L. Miles D. B. (2017). Natural selection on thermal preference, critical thermal maxima and locomotor performance. Proc. R. Soc. B Biol. Sci. 284:20170536. doi: 10.1098/rspb.2017.0536, PMID: 28814653 PMC5563794

[ref58] Ginnaw G. M. Davidson I. K. Harding H. R. Simpson S. D. Roberts N. W. Radford A. N.. (2020). Effects of multiple stressors on fish shoal collective motion are independent and vary with shoaling metric. Anim. Behav. 168, 7–17. doi: 10.1016/j.anbehav.2020.07.024

[ref59] Gomez Isaza D. F. Cramp R. L. Franklin C. E. (2020). Simultaneous exposure to nitrate and low pH reduces the blood oxygen-carrying capacity and functional performance of a freshwater fish. Conservation physiol. 8:coz092. doi: 10.1093/conphys/coz092, PMID: 31988749 PMC6977012

[ref60] Granroth-Wilding H. M. Burthe S. J. Lewis S. Herborn K. A. Takahashi E. A. Daunt F.. (2015). Indirect effects of parasitism: costs of infection to other individuals can be greater than direct costs borne by the host. Proc. R. Soc. B Biol. Sci. 282:20150602. doi: 10.1098/rspb.2015.0602, PMID: 26156765 PMC4528545

[ref61] Grigaltchik V. S. Ward A. J. W. Seebacher F. (2012). Thermal acclimation of interactions: differential responses to temperature change alter predator–prey relationship. Proc. R. Soc. B Biol. Sci. 279, 4058–4064. doi: 10.1098/rspb.2012.1277, PMID: 22859598 PMC3427582

[ref62] Guderley H. (1990). Functional significance of metabolic responses to thermal acclimation in fish muscle. Am. J. Phys. Regul. Integr. Comp. Phys. 259, R245–R252. doi: 10.1152/ajpregu.1990.259.2.R245 2201217

[ref004] Guzzo M. M. Van Leeuwen T. E. Hollins J. Koeck B. Newton M. Webber D. M.. (2018). Field testing a novel high residence positioning system for monitoring the fine-scale movements of aquatic organisms. Methods Ecol. Evol. 9, 1478–1488. 30008993 10.1111/2041-210X.12993 PMC6033000

[ref63] Hack M. A. Thompson D. J. Fernandes D. M. (1997). Fighting in males of the autumn spider, Metellina segmentata: effects of relative body size, prior residency and female value on contest outcome and duration. Ethology 103, 488–498. doi: 10.1111/j.1439-0310.1997.tb00162.x

[ref64] Hansen M. J. Ligocki I. Y. Zillig K. E. Steel A. E. Todgham A. E. Fangue N. A. (2020). Risk-taking and locomotion in foraging threespine sticklebacks (*Gasterosteus aculeatus*): the effect of nutritional stress is dependent on social context. Behav. Ecol. Sociobiol. 74:12. doi: 10.1007/s00265-019-2795-4

[ref65] Hasenjager M. J. Dugatkin L. A. (2017). Familiarity affects network structure and information flow in guppy (*Poecilia reticulata*) shoals. Behav. Ecol. 28, 233–242. doi: 10.1093/beheco/arw152

[ref66] Hasenjager M. J. Hoppitt W. Dugatkin L. A. (2020). Personality composition determines social learning pathways within shoaling fish. Proc. R. Soc. B Biol. Sci. 287:20201871. doi: 10.1098/rspb.2020.1871, PMID: 33023411 PMC7657854

[ref67] Hawley D. M. Etienne R. S. Ezenwa V. O. Jolles A. E. (2011). Does animal behavior underlie covariation between hosts’ exposure to infectious agents and susceptibility to infection? Implications for disease dynamics. Integr. Comp. Biol. 51, 528–539. doi: 10.1093/icb/icr062, PMID: 21700577

[ref68] Heinze J. Walter B. (2010). Moribund ants leave their nests to die in social isolation. Curr. Biol. 20, 249–252. doi: 10.1016/j.cub.2009.12.031, PMID: 20116243

[ref69] Herskin J. Steffensen J. F. (1998). Energy savings in sea bass swimming in a school: measurements of tail beat frequency and oxygen consumption at different swimming speeds. J. Fish Biol. 53, 366–376. doi: 10.1111/j.1095-8649.1998.tb00986.x

[ref70] Hock K. Huber R. (2009). Models of winner and loser effects: a cost-benefit analysis. Behaviour 146, 69–87. doi: 10.1163/156853908X390931

[ref71] Huang Y. Fu S. Cooke S. J. Xia J. (2020). Is repeatability of metabolic rate influenced by social separation? A test with a teleost fish. Biol. Lett. 16:20190825. doi: 10.1098/rsbl.2019.0825, PMID: 32343938 PMC7211464

[ref005] Huffman M. A. Chapman C. A. (2009). Primate parasite ecology: the dynamics and study of host-parasite relationships. Cambridge, UK: Cambridge University Press.

[ref72] Hughes D. P. Kathirithamby J. Turillazzi S. Beani L. (2004). Social wasps desert the colony and aggregate outside if parasitized: parasite manipulation? Behav. Ecol. 15, 1037–1043. doi: 10.1093/beheco/arh111

[ref74] I’Anson Price R. Dulex N. Vial N. Vincent C. Grüter C. (2019). Honeybees forage more successfully without the “dance language” in challenging environments. Scientific Advan. 5:eaat0450. doi: 10.1126/sciadv.aat0450 PMC6374110 30788430

[ref75] Ioannou C. C. (2017). Swarm intelligence in fish? The difficulty in demonstrating distributed and self-organised collective intelligence in (some) animal groups. Behav. Process. 141, 141–151. doi: 10.1016/j.beproc.2016.10.005, PMID: 27737770

[ref76] Ioannou C. C. Rocque F. Herbert-Read J. E. Duffield C. Firth J. A. (2019). Predators attacking virtual prey reveal the costs and benefits of leadership. Proc. Natl. Acad. Sci. 116, 8925–8930. doi: 10.1073/pnas.1816323116, PMID: 30988193 PMC6500137

[ref77] Ioannou C. C. Singh M. Couzin I. D. (2015). Potential leaders trade off goal-oriented and socially oriented behavior in mobile animal groups. Am. Nat. 186, 284–293. doi: 10.1086/681988, PMID: 26655156

[ref78] Jablonszky M. Szász E. Markó G. Török J. Herczeg G. Garamszegi L. Z. (2017). Escape ability and risk-taking behaviour in a Hungarian population of the collared flycatcher (Ficedula albicollis). Behav. Ecol. Sociobiol. 71, 1–12. doi: 10.1007/s00265-017-2276-6

[ref79] Jeschke J. M. Tollrian R. (2007). Prey swarming: which predators become confused and why? Anim. Behav. 74, 387–393. doi: 10.1016/j.anbehav.2006.08.020

[ref80] Johnson T. Bennett A. (1995). The thermal acclimation of burst escape performance in fish: an integrated study of molecular and cellular physiology and organismal performance. J. Exp. Biol. 198, 2165–2175. doi: 10.1242/jeb.198.10.2165 9320080

[ref81] Jolles J. W. (2021). Broad-scale applications of the raspberry pi: A review and guide for biologists. Methods Ecol. Evol. 12, 1562–1579. doi: 10.1111/2041-210X.13652

[ref82] Jolles J. W. Boogert N. J. Sridhar V. H. Couzin I. D. Manica A. (2017). Consistent individual differences drive collective behavior and group functioning of schooling fish. Curr. Biol. 27, 2862–2868.e7. doi: 10.1016/j.cub.2017.08.004 28889975 PMC5628957

[ref83] Jolles J. W. King A. J. Killen S. S. (2020a). The role of individual heterogeneity in collective animal behaviour. Trends Ecol. Evol. 35, 278–291. doi: 10.1016/j.tree.2019.11.001 31879039

[ref84] Jolles J. W. Mazué G. P. Davidson J. Behrmann-Godel J. Couzin I. D. (2020c). Schistocephalus parasite infection alters sticklebacks’ movement ability and thereby shapes social interactions. Sci. Rep. 10, 1–11. doi: 10.1038/s41598-020-69057-0 32703965 PMC7378215

[ref85] Jolles J. W. Weimar N. Landgraf T. Romanczuk P. Krause J. Bierbach D. (2020b). Group-level patterns emerge from individual speed as revealed by an extremely social robotic fish. Biol. Lett. 16:20200436. doi: 10.1098/rsbl.2020.0436 32933404 PMC7532714

[ref86] Joyce W. Ozolina K. Mauduit F. Ollivier H. Claireaux G. Shiels H. A. (2016). Individual variation in whole-animal hypoxia tolerance is associated with cardiac hypoxia tolerance in a marine teleost. Biol. Lett. 12:20150708. doi: 10.1098/rsbl.2015.0708, PMID: 26740561 PMC4785915

[ref87] Jutfelt F. Norin T. Ern R. Overgaard J. Wang T. McKenzie D. J.. (2018). Oxygen- and capacity-limited thermal tolerance: blurring ecology and physiology. J. Exp. Biol. 221:jeb169615. doi: 10.1242/jeb.169615 29321291

[ref88] Kerth G. Ebert C. Schmidtke C. (2006). Group decision making in fission–fusion societies: evidence from two-field experiments in Bechstein’s bats. Proc. R. Soc. B Biol. Sci. 273, 2785–2790. doi: 10.1098/rspb.2006.3647, PMID: 17015328 PMC1635504

[ref89] Killen S. S. Adriaenssens B. Marras S. Claireaux G. Cooke S. J. (2016a). Context dependency of trait repeatability and its relevance for management and conservation of fish populations. Conservation Physiol. 4:cow007. doi: 10.1093/conphys/cow007, PMID: 27382470 PMC4922260

[ref90] Killen S. S. Calsbeek R. Williams T. D. (2017a). The ecology of exercise: mechanisms underlying individual variation in behavior, activity, and performance: an introduction to symposium. Integr. Comp. Biol. 57, 185–194. doi: 10.1093/icb/icx083, PMID: 28859409 PMC5886314

[ref91] Killen S. S. Fu C. Wu Q. Wang Y.-X. Fu S.-J. (2016b). The relationship between metabolic rate and sociability is altered by food deprivation. Funct. Ecol. 30, 1358–1365. doi: 10.1111/1365-2435.12634

[ref92] Killen S. S. Marras S. McKenzie D. J. (2011). Fuel, fasting, fear: routine metabolic rate and food deprivation exert synergistic effects on risk-taking in individual juvenile European sea bass. J. Anim. Ecol. 80, 1024–1033. doi: 10.1111/j.1365-2656.2011.01844.x 21790592

[ref93] Killen S. S. Marras S. Metcalfe N. B. McKenzie D. J. Domenici P. (2013). Environmental stressors alter relationships between physiology and behaviour. Trends Ecol. Evol. 28, 651–658. doi: 10.1016/j.tree.2013.05.005 23756106

[ref94] Killen S. S. Marras S. Nadler L. Domenici P. (2017b). The role of physiological traits in assortment among and within fish shoals. Philosophic. Trans. Royal Soc. B Biologic. Sci. 372:20160233. doi: 10.1098/rstb.2016.0233, PMID: 28673911 PMC5498295

[ref95] Killen S. S. Marras S. Ryan M. R. Domenici P. McKenzie D. J. (2012b). A relationship between metabolic rate and risk-taking behaviour is revealed during hypoxia in juvenile European sea bass. Funct. Ecol. 26, 134–143. doi: 10.1111/j.1365-2435.2011.01920.x

[ref96] Killen S. S. Marras S. Steffensen J. F. McKenzie D. J. (2012a). Aerobic capacity influences the spatial position of individuals within fish schools. Proc. R. Soc. B Biol. Sci. 279, 357–364. doi: 10.1098/rspb.2011.1006, PMID: 21653593 PMC3223687

[ref97] Killen S. S. Mitchell M. D. Rummer J. L. Chivers D. P. Ferrari M. C. O. Meekan M. G.. (2014). Aerobic scope predicts dominance during early life in a tropical damselfish. Funct. Ecol. 28, 1367–1376. doi: 10.1111/1365-2435.12296

[ref98] Killen S. S. Nadler L. E. Grazioso K. Cox A. McCormick M. I. (2021). The effect of metabolic phenotype on sociability and social group size preference in a coral reef fish. Ecol. Evol. 11, 8585–8594. doi: 10.1002/ece3.7672

[ref99] King A. J. Douglas C. M. S. Huchard E. Isaac N. J. B. Cowlishaw G. (2008). Dominance and affiliation mediate despotism in a social primate. Curr. Biol. 18, 1833–1838. doi: 10.1016/j.cub.2008.10.048, PMID: 19026539

[ref100] Kingsolver J. Diamond S. Gomulkiewicz R. (2014). “Curve-thinking: understanding reaction norms and developmental trajectories as traits,” in Integrative Organismal Biology. eds. Martin L. B. Ghalambor C. K. Woods H. A. (New Jersey: John Wiley & Sons Ltd.), 39–53.

[ref101] Kingsolver J. G. Gomulkiewicz R. (2003). Environmental variation and selection on performance curves. Integr. Comp. Biol. 43, 470–477. doi: 10.1093/icb/43.3.470, PMID: 21680455

[ref102] Klein S. Cabirol A. Devaud J.-M. Barron A. B. Lihoreau M. (2017). Why bees are so vulnerable to environmental stressors. Trends Ecol. Evol. 32, 268–278. doi: 10.1016/j.tree.2016.12.009 28111032

[ref103] Kochhann D. (2017). Social hierarchy and resting metabolic rate in the dwarf cichlid Apistogramma agassizii: the role of habitat enrichment. Hydrobiologia 789, 123–131. doi: 10.1007/s10750-016-2806-7

[ref104] Kochhann D. Campos D. F. Val A. L. (2015). Experimentally increased temperature and hypoxia affect stability of social hierarchy and metabolism of the Amazonian cichlid Apistogramma agassizii. Comp. Biochem. Physiol. A Mol. Integr. Physiol. 190, 54–60. doi: 10.1016/j.cbpa.2015.09.006 26387464

[ref105] Kondo J. Downes S. J. (2007). Does social behaviour reliably reflect temperature-dependent physiological capacity in geckos? Anim. Behav. 74, 873–880. doi: 10.1016/j.anbehav.2006.10.030

[ref106] Krause J. Hoare D. J. Croft D. Lawrence J. Ward A. Ruxton G. D.. (2000). Fish shoal composition: mechanisms and constraints. *Proceedings of the Royal Society of London*. Series B Biologic. Sci. 267, 2011–2017. doi: 10.1098/rspb.2000.1243, PMID: 11075715 PMC1690773

[ref107] Krause J. Ruxton G. D. (2002). Living in Groups. New York: Oxford University Press.

[ref108] Kurtz J. Wiesner A. Götz P. Sauer K. P. (2000). Gender differences and individual variation in the immune system of the scorpionfly *Panorpa vulgaris* (Insecta: Mecoptera). Dev. Comp. Immunol. 24, 1–12. doi: 10.1016/S0145-305X(99)00057-9 10689094

[ref109] Larcombe S. D. Bedhomme S. Garnier S. Cellier-Holzem E. Faivre B. Sorci G. (2013). Social interactions modulate the virulence of avian malaria infection. Int. J. Parasitol. 43, 861–867. doi: 10.1016/j.ijpara.2013.05.008, PMID: 23792297

[ref110] Lefevre S. (2016). Are global warming and ocean acidification conspiring against marine ectotherms? A meta-analysis of the respiratory effects of elevated temperature, high CO2 and their interaction. Conservation Physiol. 4:cow009. doi: 10.1093/conphys/cow009, PMID: 27382472 PMC4922249

[ref111] Lennox R. J. Crook D. A. Moyle P. B. Struthers D. P. Cooke S. J. (2019). Toward a better understanding of freshwater fish responses to an increasingly drought-stricken world. Rev. Fish Biol. Fish. 29, 71–92. doi: 10.1007/s11160-018-09545-9

[ref112] MacGregor H. E. A. Cottage A. Ioannou C. C. (2021). Suppression of personality variation in boldness during foraging in three-spined sticklebacks. Behav. Ecol. Sociobiol. 75:71. doi: 10.1007/s00265-021-03007-2

[ref113] MacGregor H. E. A. Herbert-Read J. E. Ioannou C. C. (2020). Information can explain the dynamics of group order in animal collective behaviour. Nat. Commun. 11:2737. doi: 10.1038/s41467-020-16578-x, PMID: 32483141 PMC7264142

[ref114] Magoulick D. D. Kobza R. M. (2003). The role of refugia for fishes during drought: a review and synthesis. Freshw. Biol. 48, 1186–1198. doi: 10.1046/j.1365-2427.2003.01089.x

[ref115] Maierdiyali A. Wang L. Luo Y. Li Z. (2020). Effect of tank size on zebrafish behavior and physiology. Animals 10:2353. doi: 10.3390/ani10122353, PMID: 33317187 PMC7763847

[ref116] Makrinos D. L. Bowden T. J. (2016). Natural environmental impacts on teleost immune function. Fish Shellfish Immunol. 53, 50–57. doi: 10.1016/j.fsi.2016.03.008 26973022

[ref117] Marras S. Claireaux G. McKenzie D. J. Nelson J. A. (2010). Individual variation and repeatability in aerobic and anaerobic swimming performance of European sea bass, *Dicentrarchus labrax*. J. Exp. Biol. 213, 26–32. doi: 10.1242/jeb.032136 20008358

[ref118] Marras S. Killen S. S. Lindström J. McKenzie D. J. Steffensen J. F. Domenici P. (2015). Fish swimming in schools save energy regardless of their spatial position. Behav. Ecol. Sociobiol. 69, 219–226. doi: 10.1007/s00265-014-1834-4, PMID: 25620833 PMC4293471

[ref119] Martin J. G. A. Nussey D. H. Wilson A. J. Réale D. (2011). Measuring individual differences in reaction norms in field and experimental studies: a power analysis of random regression models. Methods Ecol. Evol. 2, 362–374. doi: 10.1111/j.2041-210X.2010.00084.x

[ref120] Mas-Martí E. García-Berthou E. Sabater S. Tomanova S. Muñoz I. (2010). “Comparing fish assemblages and trophic ecology of permanent and intermittent reaches in a mediterranean stream,” in Global Change and River Ecosystems—Implications for Structure, Function and Ecosystem Services. eds. Stevenson R. J. Sabater S. (Dordrecht: Springer Netherlands), 167–180.

[ref121] Mathot K. J. Dekinga A. Piersma T. (2017). An experimental test of state–behaviour feedbacks: gizzard mass and foraging behaviour in red knots. Funct. Ecol. 31, 1111–1121. doi: 10.1111/1365-2435.12827

[ref122] Mathot K. J. Dingemanse N. J. Nakagawa S. (2019). The covariance between metabolic rate and behaviour varies across behaviours and thermal types: meta-analytic insights. Biol. Rev. 94, 1056–1074. doi: 10.1111/brv.12491, PMID: 30588731

[ref123] Mccarthy I. D. (2001). Competitive ability is related to metabolic asymmetry in juvenile rainbow trout. J. Fish Biol. 59, 1002–1014. doi: 10.1111/j.1095-8649.2001.tb00167.x

[ref124] McComb K. Moss C. Durant S. M. Baker L. Sayialel S. (2001). Matriarchs as repositories of social knowledge in african elephants. Science 292, 491–494. doi: 10.1126/science.1057895, PMID: 11313492

[ref125] McCune K. Jablonski P. Lee S. Ha R. (2018). Evidence for personality conformity, not social niche specialization in social jays. Behav. Ecol. 29, 910–917. doi: 10.1093/beheco/ary055

[ref126] McKenzie D. J. Garofalo E. Winter M. J. Ceradini S. Verweij F. Day N.. (2007). Complex physiological traits as biomarkers of the sub-lethal toxicological effects of pollutant exposure in fishes. Philosophic. Trans. Royal Soc. B Biologic. Sci. 362, 2043–2059. doi: 10.1098/rstb.2007.2100, PMID: 17475615 PMC2442853

[ref127] McLean S. Persson A. Norin T. Killen S. S. (2018). Metabolic costs of feeding predictively alter the spatial distribution of individuals in fish schools. Curr. Biol. 28, 1144–1149.e4. doi: 10.1016/j.cub.2018.02.043 29576472

[ref128] McNett G. D. Luan L. H. Cocroft R. B. (2010). Wind-induced noise alters signaler and receiver behavior in vibrational communication. Behav. Ecol. Sociobiol. 64, 2043–2051. doi: 10.1007/s00265-010-1018-9

[ref129] Meager J. J. Domenici P. Shingles A. Utne-Palm A. C. (2006). Escape responses in juvenile Atlantic cod *Gadus morhua L*.: the effects of turbidity and predator speed. J. Exp. Biol. 209, 4174–4184. doi: 10.1242/jeb.02489 17023610

[ref130] Metcalfe N. B. Van Leeuwen T. E. Killen S. S. (2016). Does individual variation in metabolic phenotype predict fish behaviour and performance? J. Fish Biol. 88, 298–321. doi: 10.1111/jfb.12699, PMID: 26577442 PMC4991269

[ref131] Michelangeli M. Goulet C. T. Kang H. S. Wong B. B. M. Chapple D. G. (2018). Integrating thermal physiology within a syndrome: locomotion, personality and habitat selection in an ectotherm. Funct. Ecol. 32, 970–981. doi: 10.1111/1365-2435.13034

[ref132] Miln C. Ward A. J. Seebacher F. (2021). Social rank and not physiological capacity determines competitive success in zebrafish (Danio rerio). R. Soc. Open Sci. 8:210146. doi: 10.1098/rsos.210146, PMID: 33868699 PMC8025299

[ref006] Montiglio P. O. Ferrari C. Reale D. (2013). Social niche specialization under constraints: personality, social interactions and environmental heterogeneity. Philos. Trans. R. Soc. B, Biol. Sci. 368:20120343. 10.1098/rstb.2012.0343 PMC3638446 23569291

[ref133] Moyers S. C. Adelman J. S. Farine D. R. Moore I. T. Hawley D. M. (2018). Exploratory behavior is linked to stress physiology and social network centrality in free-living house finches (*Haemorhous mexicanus*). Horm. Behav. 102, 105–113. doi: 10.1016/j.yhbeh.2018.05.005, PMID: 29758182

[ref134] Mueller T. O’Hara R. B. Converse S. J. Urbanek R. P. Fagan W. F. (2013). Social Learning of migratory performance. Science 341, 999–1002. doi: 10.1126/science.1237139, PMID: 23990559

[ref135] Munson A. Michelangeli M. Sih A. (2021). Stable social groups foster conformity and among-group differences. Anim. Behav. 174, 197–206. doi: 10.1016/j.anbehav.2021.02.011

[ref136] Murren C. J. Maclean H. J. Diamond S. E. Steiner U. K. Heskel M. A. Handelsman C. A.. (2014). Evolutionary change in continuous reaction norms. Am. Nat. 183, 453–467. doi: 10.1086/675302, PMID: 24642491

[ref137] Nagy M. Ákos Z. Biro D. Vicsek T. (2010). Hierarchical group dynamics in pigeon flocks. Nature 464, 890–893. doi: 10.1038/nature08891, PMID: 20376149

[ref138] Nati J. J. H. Lindström J. Halsey L. G. Killen S. S. (2016). Is there a trade-off between peak performance and performance breadth across temperatures for aerobic scope in teleost fishes? Biol. Lett. 12:20160191. doi: 10.1098/rsbl.2016.0191, PMID: 27677812 PMC5046912

[ref139] Navas C. James R. Wakeling J. Kemp K. Johnston I. (1999). An integrative study of the temperature dependence of whole animal and muscle performance during jumping and swimming in the frog *Rana temporaria*. J. Comp. Physiol. B 169, 588–596. doi: 10.1007/s003600050259 10633564

[ref141] Nieman C. L. Gray S. M. (2019). Visual performance impaired by elevated sedimentary and algal turbidity in walleye Sander vitreus and emerald shiner *Notropis atherinoides*. J. Fish Biol. 95, 186–199. doi: 10.1111/jfb.13878, PMID: 30511351

[ref142] Norin T. Clark T. D. (2017). Fish face a trade-off between ‘eating big’ for growth efficiency and ‘eating small’ to retain aerobic capacity. Biol. Lett. 13:20170298. doi: 10.1098/rsbl.2017.0298, PMID: 28931728 PMC5627169

[ref143] Norin T. Metcalfe N. B. (2019). Ecological and evolutionary consequences of metabolic rate plasticity in response to environmental change. Philosophic. Trans. Royal Society B Biologic. Sci. 374:20180180. doi: 10.1098/rstb.2018.0180, PMID: 30966964 PMC6365862

[ref144] Nowakowski A. J. Peaden J. M. Tuberville T. D. Buhlmann K. A. Todd B. D. (2020). Thermal performance curves based on field movements reveal context-dependence of thermal traits in a desert ectotherm. Landsc. Ecol. 35, 893–906. doi: 10.1007/s10980-020-00986-x

[ref145] Ord T. J. Stamps J. A. (2017). Why does the rate of signal production in ectotherms vary with temperature? Behav. Ecol. 28, 1272–1282. doi: 10.1093/beheco/arx089

[ref146] Pang X. Fu S.-J. Zhang Y.-G. (2015). Individual variation in metabolism and swimming performance in juvenile black carp (*Mylopharyngodon piceus*) and the effects of hypoxia. Mar. Freshw. Behav. Physiol. 48, 431–443. doi: 10.1080/10236244.2015.1090205

[ref147] Patterson J. E. Ruckstuhl K. E. (2013). Parasite infection and host group size: a meta-analytical review. Parasitology 140, 803–813. doi: 10.1017/S0031182012002259, PMID: 23425516 PMC3638372

[ref148] Pettit B. Akos Z. Vicsek T. Biro D. (2015). Speed determines leadership and leadership determines learning during pigeon flocking. Curr. Biol. 25, 3132–3137. doi: 10.1016/j.cub.2015.10.044, PMID: 26628007

[ref149] Pineda M. Aragao I. McKenzie D. J. Killen S. S. (2020). Social dynamics obscure the effect of temperature on air breathing in Corydoras catfish. J. Exp. Biol. 223:jeb222133. doi: 10.1242/jeb.222133 33097572 PMC7673363

[ref150] Poirotte C. Massol F. Herbert A. Willaume E. Bomo P. M. Kappeler P. M.. (2017). Mandrills use olfaction to socially avoid parasitized conspecifics. Sci. Adv. 3:e1601721. doi: 10.1126/sciadv.1601721, PMID: 28435875 PMC5384805

[ref151] Pörtner H. O. (2010). Oxygen-and capacity-limitation of thermal tolerance: a matrix for integrating climate-related stressor effects in marine ecosystems. J. Exp. Biol. 213, 881–893. doi: 10.1242/jeb.037523 20190113

[ref152] Pörtner H. O. Farrell A. P. (2008). Physiology and climate change. Science 322, 690–692. doi: 10.1126/science.1163156, PMID: 18974339

[ref153] Radford A. N. Kerridge E. Simpson S. D. (2014). Acoustic communication in a noisy world: can fish compete with anthropogenic noise? Behav. Ecol. 25, 1022–1030. doi: 10.1093/beheco/aru029

[ref154] Ranta E. Rita H. Lindstrom K. (1993). Competition vs. cooperation: success of individuals foraging alone and in groups. Am. Nat. 142, 42–58. doi: 10.1086/285528, PMID: 19425970

[ref155] Raulo A. Ruokolainen L. Lane A. Amato K. Knight R. Leigh S.. (2018). Social behaviour and gut microbiota in red-bellied lemurs (*Eulemur rubriventer*): In search of the role of immunity in the evolution of sociality. J. Anim. Ecol. 87, 388–399. doi: 10.1111/1365-2656.12781 29205327

[ref157] Roche D. G. Careau V. Binning S. A. (2016). Demystifying animal ‘personality’ (or not): why individual variation matters to experimental biologists. J. Exp. Biol. 219, 3832–3843. doi: 10.1242/jeb.146712, PMID: 27852750

[ref158] Rodgers G. M. Downing B. Morrell L. J. (2015). Prey body size mediates the predation risk associated with being “odd”. Behav. Ecol. 26, 242–246. doi: 10.1093/beheco/aru185

[ref160] Ross R. M. (1978). Territorial behavior and ecology of the anemonefish *Amphiprion melanopus* on Guam 1. Z. Tierpsychol. 46, 71–83. doi: 10.1111/j.1439-0310.1978.tb01439.x

[ref161] Ruckstuhl K. Neuhaus P. (2006). Sexual Segregation in Vertebrates. New York: Cambridge University Press.

[ref162] Sankey D. W. E. Shepard E. L. C. Biro D. Portugal S. J. (2019). Speed consensus and the ‘goldilocks principle’ in flocking birds (Columba livia). Anim. Behav. 157, 105–119. doi: 10.1016/j.anbehav.2019.09.001

[ref163] Schaerf T. M. Dillingham P. W. Ward A. J. W. (2017). The effects of external cues on individual and collective behavior of shoaling fish. Sci. Adv. 3:e1603201. doi: 10.1126/sciadv.1603201, PMID: 28691088 PMC5482554

[ref164] Schmitz R. A. Baldassarre G. A. (1992). Contest asymmetry and multiple bird conflicts during foraging among nonbreeding American flamingos in Yucatan, Mexico. Condor 94, 254–259. doi: 10.2307/1368814

[ref165] Seebacher F. Krause J. (2017). Physiological mechanisms underlying animal social behaviour. Philosophic. Trans. Royal Society B Biologic. Sci. 372:20160231. doi: 10.1098/rstb.2016.0231, PMID: 28673909 PMC5498293

[ref166] Sheets C. N. Schmidt D. R. Hurtado P. J. Byrne A. Q. Rosenblum E. B. Richards-Zawacki C. L.. (2021). Thermal performance curves of multiple isolates of batrachochytrium dendrobatidis, a lethal pathogen of amphibians. Front. Vet. Sci. 8:648. doi: 10.3389/fvets.2021.687084 PMC8258153 34239916

[ref167] Sih A. (2013). Understanding variation in behavioural responses to human-induced rapid environmental change: a conceptual overview. Anim. Behav. 85, 1077–1088. doi: 10.1016/j.anbehav.2013.02.017

[ref168] Sih A. Mathot K. J. Moirón M. Montiglio P.-O. Wolf M. Dingemanse N. J. (2015). Animal personality and state–behaviour feedbacks: a review and guide for empiricists. Trends Ecol. Evol. 30, 50–60. doi: 10.1016/j.tree.2014.11.004 25498413

[ref169] Simons A. M. (2004). Many wrongs: the advantage of group navigation. Trends Ecol. Evol. 19, 453–455. doi: 10.1016/j.tree.2004.07.001 16701304

[ref170] Sneddon L. U. Huntingford F. A. Taylor A. C. (1998). Impact of an ecological factor on the costs of resource acquisition: fighting and metabolic physiology of crabs. Funct. Ecol. 12, 808–815. doi: 10.1046/j.1365-2435.1998.00249.x

[ref171] Snyder-Mackler N. Sanz J. Kohn J. N. Brinkworth J. F. Morrow S. Shaver A. O.. (2016). Social status alters immune regulation and response to infection in macaques. Science 354, 1041–1045. doi: 10.1126/science.aah3580, PMID: 27885030 PMC5498102

[ref172] Sogard S. M. Olla B. L. (1997). The influence of hunger and predation risk on group cohesion in a pelagic fish, walleye Pollock *Theragra chalcogramma*. Environ. Biol. Fish 50, 405–413. doi: 10.1023/A:1007393307007

[ref173] Spencer K. A. (2017). Developmental stress and social phenotypes: integrating neuroendocrine, behavioural and evolutionary perspectives. Philosophic. Trans. Royal Society B Biologic. Sci. 372:20160242. doi: 10.1098/rstb.2016.0242, PMID: 28673918 PMC5498302

[ref174] Spiegel O. Leu S. T. Sih A. Godfrey S. S. Bull C. M. (2015). When the going gets tough: behavioural type-dependent space use in the sleepy lizard changes as the season dries. Proc. R. Soc. B Biol. Sci. 282:20151768. doi: 10.1098/rspb.2015.1768, PMID: 26609082 PMC4685807

[ref175] Takada H. Minami M. (2021). Open habitats promote female group formation in a solitary ungulate: the Japanese serow. Behav. Ecol. Sociobiol. 75:60. doi: 10.1007/s00265-021-02999-1

[ref176] Teichroeb J. A. Sicotte P. (2018). Cascading competition: the seasonal strength of scramble influences between-group contest in a folivorous primate. Behav. Ecol. Sociobiol. 72:6. doi: 10.1007/s00265-017-2418-x

[ref177] Theodorakis C. W. (1989). Size segregation and the effects of oddity on predation risk in minnow schools. Anim. Behav. 38, 496–502. doi: 10.1016/S0003-3472(89)80042-9

[ref178] Traniello J. F. Rosengaus R. B. Savoie K. (2002). The development of immunity in a social insect: evidence for the group facilitation of disease resistance. Proc. Natl. Acad. Sci. 99, 6838–6842. doi: 10.1073/pnas.102176599, PMID: 12011442 PMC124490

[ref179] Turbill C. Ruf T. Rothmann A. Arnold W. (2013). Social dominance is associated with individual differences in heart rate and energetic response to food restriction in female Red Deer. Physiol. Biochem. Zool. 86, 528–537. doi: 10.1086/672372, PMID: 23995483

[ref180] Ugelvig L. V. Cremer S. (2007). Social prophylaxis: group interaction promotes collective immunity in ant colonies. Curr. Biol. 17, 1967–1971. doi: 10.1016/j.cub.2007.10.029, PMID: 17980590

[ref181] van Berkum F. H. (1988). Latitudinal patterns of the thermal sensitivity of sprint speed in lizards. Am. Nat. 132, 327–343. doi: 10.1086/284856 28556314

[ref182] van de Pol M. (2012). Quantifying individual variation in reaction norms: how study design affects the accuracy, precision and power of random regression models. Methods Ecol. Evol. 3, 268–280. doi: 10.1111/j.2041-210X.2011.00160.x

[ref183] von Frisch K. (1967). The Dance Language and Orientation of Bees. Harvard, USA: Harvard University Press.

[ref184] Wade A. S. I. Ramnarine I. W. Ioannou C. C. (2020). The effect of group size on the speed of decision making depends on compromise and predation risk across populations in the guppy *Poecilia reticulata*. Behaviour 157, 1173–1192. doi: 10.1163/1568539X-bja10044

[ref185] Walsberg G. E. Lea M. S. Hillman S. S. (1986). Individual variation in maximum aerobic capacity: cardiovascular and enzymatic correlates in *Rana catesbeiana*. J. Exp. Zool. 239, 1–5. doi: 10.1002/jez.1402390102 3489066

[ref186] Ward A. J. W. Herbert-Read J. E. Schaerf T. M. Seebacher F. (2018). The physiology of leadership in fish shoals: leaders have lower maximal metabolic rates and lower aerobic scope. J. Zool. 305, 73–81. doi: 10.1111/jzo.12534

[ref187] Ward A. Webster M. (2016). “Sociality” in Sociality: The Behaviour of Group-Living Animals. eds. Ward A. Webster M. (Cham: Springer International Publishing), 1–8.

[ref188] Wascher C. A. F. Kulahci I. G. Langley E. J. G. Shaw R. C. (2018). How does cognition shape social relationships? Philosophic. Trans. Royal Society B Biologic. Sci. 373:20170293. doi: 10.1098/rstb.2017.0293, PMID: 30104437 PMC6107564

[ref189] Williams H. J. Shipley J. R. Rutz C. Wikelski M. Wilkes M. Hawkes L. A. (2021). Future trends in measuring physiology in free-living animals. Philos. Trans. R. Soc. B 376:20200230. doi: 10.1098/rstb.2020.0230, PMID: 34176330 PMC8237165

[ref190] Wilson R. S. James R. S. Kohlsdorf T. Cox V. M. (2004). Interindividual variation of isolated muscle performance and fibre-type composition in the toad *Bufo viridus*. J. Comp. Physiol. B 174, 453–459. doi: 10.1007/s00360-004-0431-7, PMID: 15185115

